# Inverse design of nanophotonic devices enabled by optimization algorithms and deep learning: recent achievements and future prospects

**DOI:** 10.1515/nanoph-2024-0536

**Published:** 2025-01-27

**Authors:** Junhyeong Kim, Jae-Yong Kim, Jungmin Kim, Yun Hyeong, Berkay Neseli, Jong-Bum You, Joonsup Shim, Jonghwa Shin, Hyo-Hoon Park, Hamza Kurt

**Affiliations:** The School of Electrical Engineering, Korea Advanced Institute of Science and Technology (KAIST), Daejeon, Republic of Korea; Department of Electrical and Computer Engineering, University of Wisconsin-Madison, Madison, WI 53706, USA; The School of Materials Science and Engineering, Korea Advanced Institute of Science and Technology (KAIST), Daejeon, Republic of Korea; National Nanofab Center (NNFC), Daejeon, Republic of Korea; John A. Paulson School of Engineering and Applied Sciences, Harvard University, Cambridge, MA, USA

**Keywords:** nanophotonics, silicon photonics, inverse design, optimization, artificial intelligence, deep learning

## Abstract

Nanophotonics, which explores significant light–matter interactions at the nanoscale, has facilitated significant advancements across numerous research fields. A key objective in this area is the design of ultra-compact, high-performance nanophotonic devices to pave the way for next-generation photonics. While conventional brute-force, intuition-based forward design methods have produced successful nanophotonic solutions over the past several decades, recent developments in optimization methods and artificial intelligence offer new potential to expand these capabilities. In this review, we delve into the latest progress in the inverse design of nanophotonic devices, where AI and optimization methods are leveraged to automate and enhance the design process. We discuss representative methods commonly employed in nanophotonic design, including various meta-heuristic algorithms such as trajectory-based, evolutionary, and swarm-based approaches, in addition to adjoint-based optimization. Furthermore, we explore state-of-the-art deep learning techniques, involving discriminative models, generative models, and reinforcement learning. We also introduce and categorize several notable inverse-designed nanophotonic devices and their respective design methodologies. Additionally, we summarize the open-source inverse design tools and commercial foundries. Finally, we provide our perspectives on the current challenges of inverse design, while offering insights into future directions that could further advance this rapidly evolving field.

## 1 Introduction

Nanophotonics, which facilitates light–matter interactions at the nanoscale, has become a major area of interest in modern optics and photonics. It can pave the way for various applications including high-speed data communications [1], data centers [2], [3], computing [4], [5], [6], [7], energy-saving [8], [9], [10], healthcare [11], [12], [13], [14], and sensing [14], [15], [16], [17], among others. To achieve these applications, it is important to design nanophotonic devices, including power splitters, grating couplers, resonators, wavelength multiplexers/de-multiplexers, modulators, switches, nanoscale lasers, photodetectors, and metasurfaces. Traditionally, devices were designed using a forward design process, where the designer determines the structural parameters based on intuition and expertise, followed by numerical calculations (e.g. finite element method [FEM], finite-difference time-domain [FDTD] method, transfer-matrix method [TMM], or rigorous coupled-wave analysis [RCWA]) to assess optical performance. Although this approach is easy to implement, intuitive, and delivers reliable results, it is often limited by high computational costs and a limited degree of freedom, which hinders the exploration of large-scale designs. In contrast, the emerging inverse design method reverses this process, overcoming these limitations. Structural design can be automatically determined by optimization algorithms or artificial intelligence (AI) based on desired optical characteristics. Inverse design methods offer a greater degree of freedom and higher potential for global optimization through an automated design process. Leveraging these advantages, several efforts in the 2000s focused on using inverse design for nanophotonic devices through optimization algorithms, leading to the discovery of high-performance devices previously unattainable [18], [19].

Recently, AI has achieved remarkable advancements across various fields such as image processing, healthcare, and material design. In many other areas of science, AI has the potential to push the boundaries of what is possible, such as by accelerating drug discovery, enhancing climate modelling, and enabling the creation of autonomous systems that can interact with complex environments. With the rapid development of AI, AI-based inverse design techniques have been widely applied to nanophotonic device design over the past decade [20], [21], [22]. From conventional deep learning methods to state-of-the-art generative models, successful demonstrations have established the potential of these design approaches.

In this article, we introduce the current progress of inverse design methods and their corresponding designs in nanophotonics. First, the theoretical details of the inverse design methods are discussed. These methods can be classified into three categories: meta-heuristic algorithm-based inverse design, adjoint methods, and AI-based inverse design. Various optimization algorithms, including trajectory-based, evolutionary, and swarm-based algorithms within meta-heuristic algorithms, as well as adjoint methods, are explored. For the AI-based inverse design algorithms, discriminative methods, generative models, and reinforcement learning techniques are discussed. Following detailed descriptions of the inverse design methods, we present selected examples of various inverse-designed nanophotonic devices, including photonic power splitters, wavelength (de)multiplexers, grating couplers, waveguide devices, and metasurfaces. We also introduce open-source inverse design tools and available fabrication facilities, which can serve as useful resources for newcomers. Finally, we address some challenges in the inverse design of nanophotonic devices, aiming to overcome obstacles in the development of future technologies.

## 2 Inverse design algorithms

Regarding the low degree of freedom in conventional forward design approaches, limitations have been encountered in optimizing device footprint and performance. In this process, numerous optimization algorithms have been implemented for the inverse design of nanophotonic devices over the past two decades. Abundant approaches including meta-heuristic algorithms, gradient-based optimization algorithms (e.g. adjoint method), and AI-based techniques, can be applied to inverse design. In the following sections, we introduce these methods along with their theoretical backgrounds and representative examples. Each of these algorithms has its own advantages and disadvantages and carefully choosing the inverse design algorithm is essential considering computational costs, the possibility of global optimization, the difficulty of implementation, etc.

### 2.1 Meta-heuristic algorithms

Meta-heuristic algorithms are problem-independent approaches that find near-optimal solutions to complex optimization problems at reasonable computational costs. These algorithms are widely applied to complex problems where an exact solution is difficult to obtain and trial-and-error approaches are impractical. In this section, several meta-heuristic algorithms commonly used for the inverse design of nanophotonic devices are investigated. Meta-heuristic algorithms can be classified as either trajectory-based or population-based, depending on the number of solutions considered during the optimization process. Furthermore, population-based algorithms can be classified as evolutionary or swarm-based, based on their exploration strategies within the search space. In this section, an overview of the several algorithms is presented. First, we introduce trajectory-based algorithms, including hill-climbing and direct binary search algorithms. Next, we discuss evolutionary algorithms, such as genetic algorithms and differential evolution. Finally, swarm-based algorithms, including ant colony optimization and particle swarm optimization, are explained.

#### 2.1.1 Trajectory-based algorithms

Trajectory algorithms explore the search space by making small incremental changes to the current solution. These algorithms follow a specific trajectory in the solution space, selecting locally optimal choices at each step. However, they do not always achieve a global optimum and can be classified as greedy algorithms. In this section, we introduce several trajectory algorithms commonly implemented for the inverse design of nanophotonic devices, namely the hill-climbing algorithm and direct binary search algorithm.

The hill-climbing algorithm is a well-known and intuitive method that begins with an initial solution and iteratively makes small changes to it to enhance it. For example, assume that there are several design parameters, such as period, width, and length. Starting from the initial guess, one of these parameters is updated, and the resulting optical performance is evaluated. If the performance improves upon the previous state, the algorithm updates the parameter and repeats the process.

Conversely, if the updated performance does not exceed the previous state, the algorithm retains the current parameter and proceeds to the next one. Consequently, all parameters are updated until the algorithm reaches a local optimum. The hill-climbing algorithm is advantageous due to its simplicity, intuitive process, and ease of implementation. Moreover, it can be readily extended with additional strategies or constraints and can efficiently find local optima when a quick solution is required. However, this method has several drawbacks. First, it often fails to find the global optimum, leading to suboptimal performance compared to other algorithms. Second, the algorithm is highly sensitive to the initial guess, which complicates the search for optimal solutions.

The direct binary search (DBS) algorithm is another well-known and intuitive method for designing nanophotonic devices. Similar to the hill-climbing algorithm, it updates the design space iteratively, but the key difference is that the DBS algorithm makes binary changes to the design parameters. It operates in a discrete search space where the design is represented in a binary format. Each element or pixel of the design can be turned on or off; for example, the state between two different materials (i.e. silicon, silicon dioxide, air, etc.) can be selected. Starting from the initial guess, the algorithm iteratively flips the state of individual pixels and evaluates the optical performance. If a change improves performance, the algorithm updates the parameter and repeats the process. If not, the algorithm retains the state and flips another pixel. As a result, all parameters are randomly updated, resulting in a locally optimized design. Its binary nature makes it particularly suited for problems where the design space is inherently discrete and non-continuous. The DBS algorithm offers a straightforward and intuitive approach to optimization, making it ideal for discrete design spaces. Since nanophotonic devices are composed of several discrete materials, the algorithm can effectively find a local optimum by making binary adjustments. However, the DBS algorithm can easily get stuck in local optima, missing the global optimum, and its performance is highly sensitive to the initial design. This method can also be computationally expensive for large design spaces due to the iterative evaluation process, and therefore may not scale well with increasing problem size or complexity.

**Figure 1:**
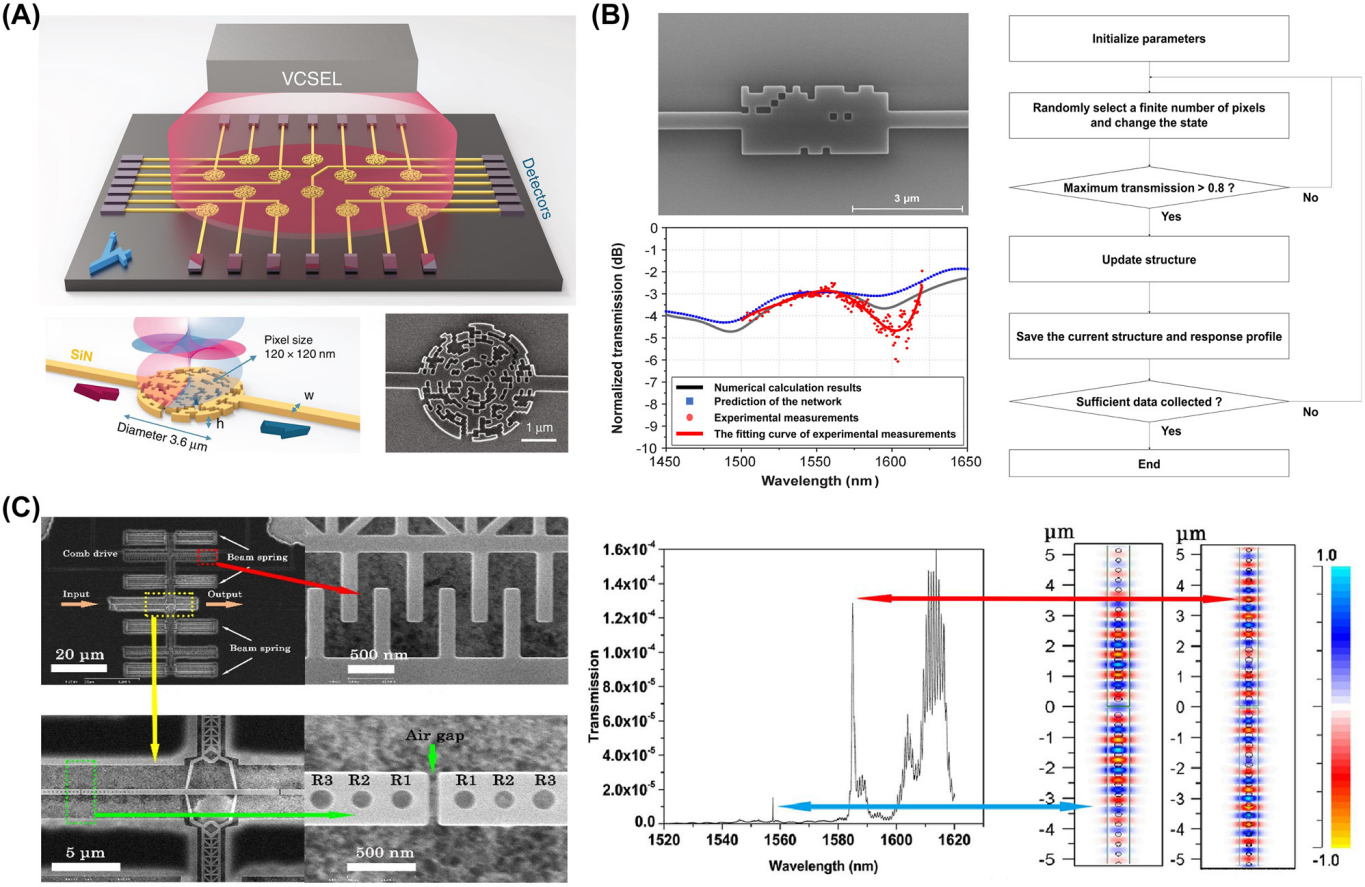
Representative examples of inverse-designed nanophotonic devices enabled by trajectory-based algorithms. (A) Schematic of the photonic spin selector inverse-designed by the direct binary search algorithm. (B) SEM image of the inverse-designed waveguide device on an SOI platform, its corresponding optical responses, and the flow chart of the trajectory-based algorithm. (C) SEM image of the tunable photonic crystal nanocavity inverse-designed by the hill-climbing algorithm (left) and corresponding optical responses (right). (A) Is reprinted from Ref. [23], with permission (CC BY 4.0); (B) is adapted from Ref. [24]; (C) is reprinted from Ref. [25], with permission from Optica Publishing Group.

Several examples of inverse-designed nanophotonic devices leveraging trajectory-based algorithms are shown in Figure 1. These successful design results demonstrate that these methods hold significant potential for designing various kinds of nanophotonic devices, especially digitized structures.

#### 2.1.2 Evolutionary algorithms

Evolutionary algorithms, inspired by principles of natural selection and genetics, are widely applied optimization techniques. These algorithms are implemented to solve complex optimization problems iteratively, by improving a set of solutions in the current generation based on mechanisms such as reproduction, mutation, selection, and recombination. Evolutionary algorithms are widely applied to inverse-design problems across various research fields and are considered powerful tools for designing nanophotonic devices. In this section, we introduce several evolutionary algorithms commonly used for the inverse design of nanophotonic devices, namely the genetic algorithm and differential evolution.

The genetic algorithm (GA) is one of the most popular evolutionary algorithms [26]. This algorithm mimics the process of natural evolution to find near-optimal solutions. In GA, each solution in the generation corresponds to a chromosome, a structured array containing the variables to be optimized. Each variable, referred to as a gene, represents a specific design parameter. In the context of photonic device design, a chromosome comprises the variables to be optimized. These parameters can be the width, radius, position, or refractive index information of the unit cells inside the design area. Therefore, a gene can correspond to a single variable within the array of a chromosome, such as the radius of a photonic crystal at a specific location. In the initial stage, GA generates a population of individuals randomly. The algorithm then evaluates the fitness of each individual in the population and selects the best solutions using methods such as roulette wheel, tournament, or rank-based selection. After selecting the individuals, the algorithm combines two parent solutions to produce offspring through a process known as crossover (or recombination). After creating offspring (child solutions), the mutation process introduces small random changes to genes to maintain genetic diversity and avoid premature convergence. After a sufficient number of generations or once satisfactory convergence of population has been achieved, the algorithm is terminated.

Differential evolution (DE) is another evolutionary algorithm similar in concept to GA [27], but particularly effective for continuous and high-dimensional optimization problems. Unlike GA, DE employs unique mechanisms for mutation and crossover, which makes it particularly well-suited for optimizing complex and nonlinear functions. In DE, the mutation process is carried out between randomly chosen vectors. The difference between two randomly chosen vectors is scaled by a mutation factor, *F*, and then added to a third randomly chosen vector, resulting in a mutant vector. Crossover in DE results in a trial vector, which is obtained by the combination between the mutant vector and a vector in the population. Each component of the trial vector is chosen from either the mutant vector or the target vector based on a predefined crossover probability, *CR*.

**Figure 2:**
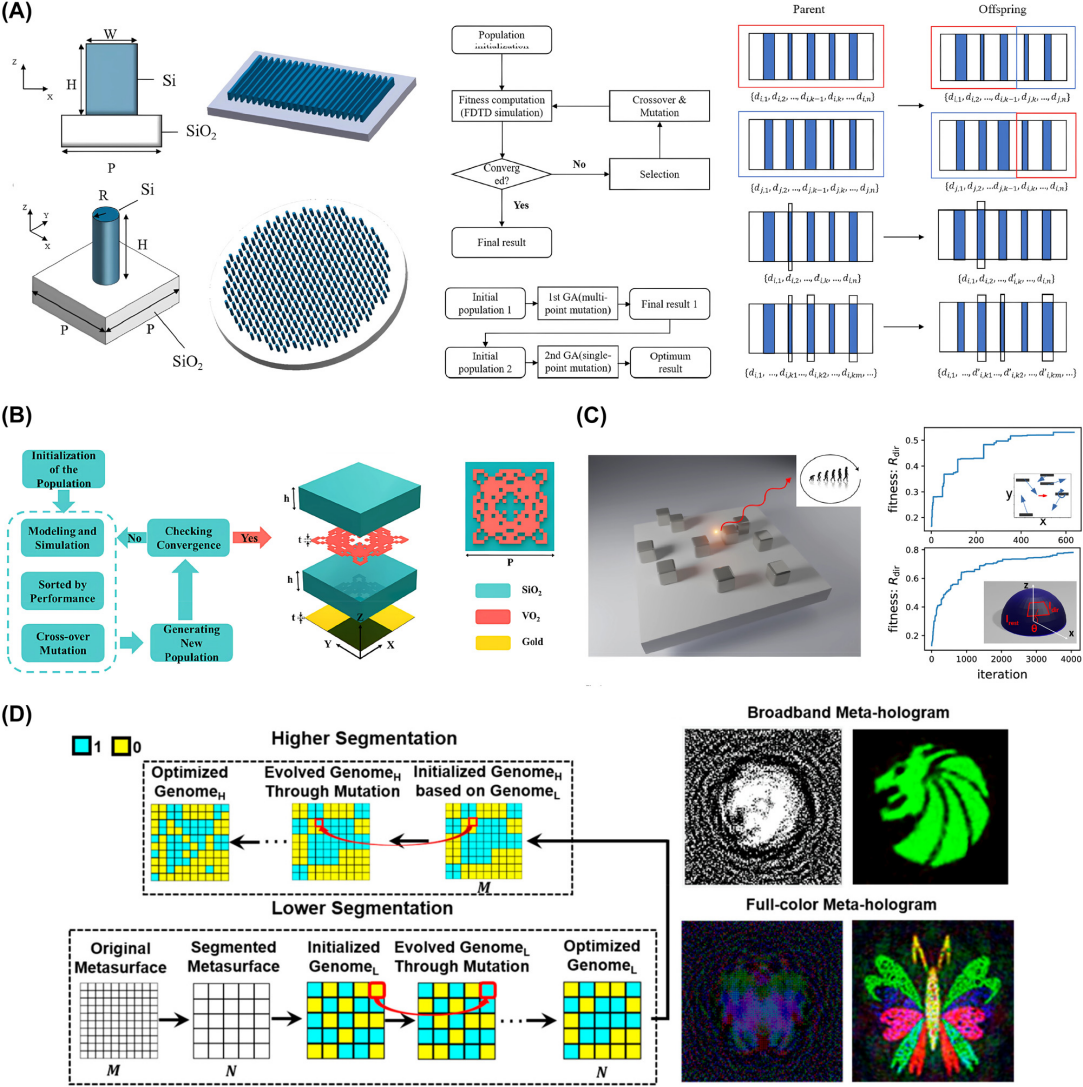
Representative examples of inverse-designed nanophotonic devices enabled by evolutionary algorithms. (A) Schematic of the inverse-designed metalens for near-infrared applications (left) and flowchart of the genetic algorithm and structural examples of the algorithm (right). (B) Flowchart of the genetic algorithm and the schematic of inverse-designed metasurface absorber. (C) Schematic of the dielectric nano-antenna inverse-designed by the differential evolution algorithm. (D) Flowchart of the segmented hierarchical evolutionary algorithm and full-color meta-hologram of optimized metasurfaces. (A) Is reprinted from Ref. [28], under the terms of the Open Access Publishing Agreement; (B) is reprinted from Ref. [29], with permission. Copyright 2024 Elsevier; (C) is reprinted from Ref. [30], with permission from Optica Publishing Group; (D) is reprinted from Ref. [31], with permission. Copyright 2024 American Chemical Society.

Several inverse-designed nanophotonic devices leveraging evolutionary algorithms are shown in Figure 2. These results highlight the potential of these methods for creating various nanophotonic devices for different applications.

#### 2.1.3 Swarm-based algorithms

Swarm-based algorithms are a class of optimization techniques inspired by the collective intelligence observed in natural systems, such as flocks of birds, schools of fish, and colonies of ants. Unlike single-agent optimization methods, swarm-based optimization methods utilize multiple agents that share information to find optimal solutions. During this process, even if some of the agents fall into local optima, the solution can reach the global optimum due to other agents. However, computational costs increase proportionally with the number of agents, which must be managed carefully based on the task. In this section, we introduce several swarm-based algorithms that are commonly implemented for the inverse design of nanophotonic devices, namely particle swarm optimization and ant colony optimization.

Particle swarm optimization (PSO) is a stochastic optimization algorithm proposed by Kennedy and Eberhart in 1995 [32]. It was inspired by the behaviour of a flock of birds: when a bird searches randomly for food, the entire flock benefits from its discoveries, thereby enhancing the group’s overall success in finding food. This concept has been applied to various optimization problems, especially for the inverse design of nanophotonic devices. During the optimization process, the structure is updated iteratively while minimizing the user-defined figure of merit (FoM), and the positions and velocities of particles in the algorithm are defined as follows:

(1) $$x_{t}=x_{t-1}+v_{t},$$

(2) $$\begin{array}{ll} v_{t}=\omega v_{t-1} & +c_{1}\eta_{1}\left(p_{\text{best,}t-1}-x_{t-1}\right)\\ & +c_{2}\eta_{2}\left(g_{\text{best,}t-1}-x_{t-1}\right), \end{array}$$

where *x*_*t*_ and *v*_*t*_ are the position and velocity of the particle at time *t*, *c*_1_ and *c*_2_ are cognitive and social constants, *η*_1_ and *η*_2_ are random coefficients, *p*_best_ and *g*_best_ represent the personal and global best, and *ω* is the inertia weight. PSO is easy to implement and capable of finding optimal solutions in high-dimensional spaces without requiring additional optimization methods (e.g. local search algorithms). Moreover, it provides a higher possibility of global optimization compared to other algorithms as the problem dimensionality increases.

Another swarm-based algorithm, ant colony optimization (ACO), is inspired by the foraging behaviour of ants in ant colonies [33]. Ants find the shortest paths to food sources by depositing pheromone trails behind them, which guides other ants to follow these paths, thereby forming efficient routes over time. By leveraging this collective behaviour, ACO effectively solves complex optimization problems. In nanophotonics, ACO can also be applied to optimize and inverse design devices. During the optimization process, an artificial ant moves from node *i* to node *j* with the probability *P*_*ij*_ defined as follows:

(3) $$P_{ij}=\frac{\tau_{ij}^{\alpha }\eta_{ij}^{\beta }}{\sum \overset{\alpha }{\underset{ij}{\tau }}\eta_{ij}^{\beta }},$$

where *τ*_*ij*_ is the total amount of pheromone deposited on the edge *i*–*j*, *η*_*ij*_ is the visibility, and *α* and *β* are the relative coefficients of the pheromone trail versus visibility. The total amount of pheromone *τ*_*ij*_ is updated with the following equation:

(4) $$\tau_{ij}=\left(1-\rho \right)\tau_{ij}+\Delta \tau_{ij},$$

where *ρ* is the pheromone evaporation coefficient within the range [0, 1], and Δ*τ*_*ij*_ is the amount of pheromone deposited by an ant.

**Figure 3:**
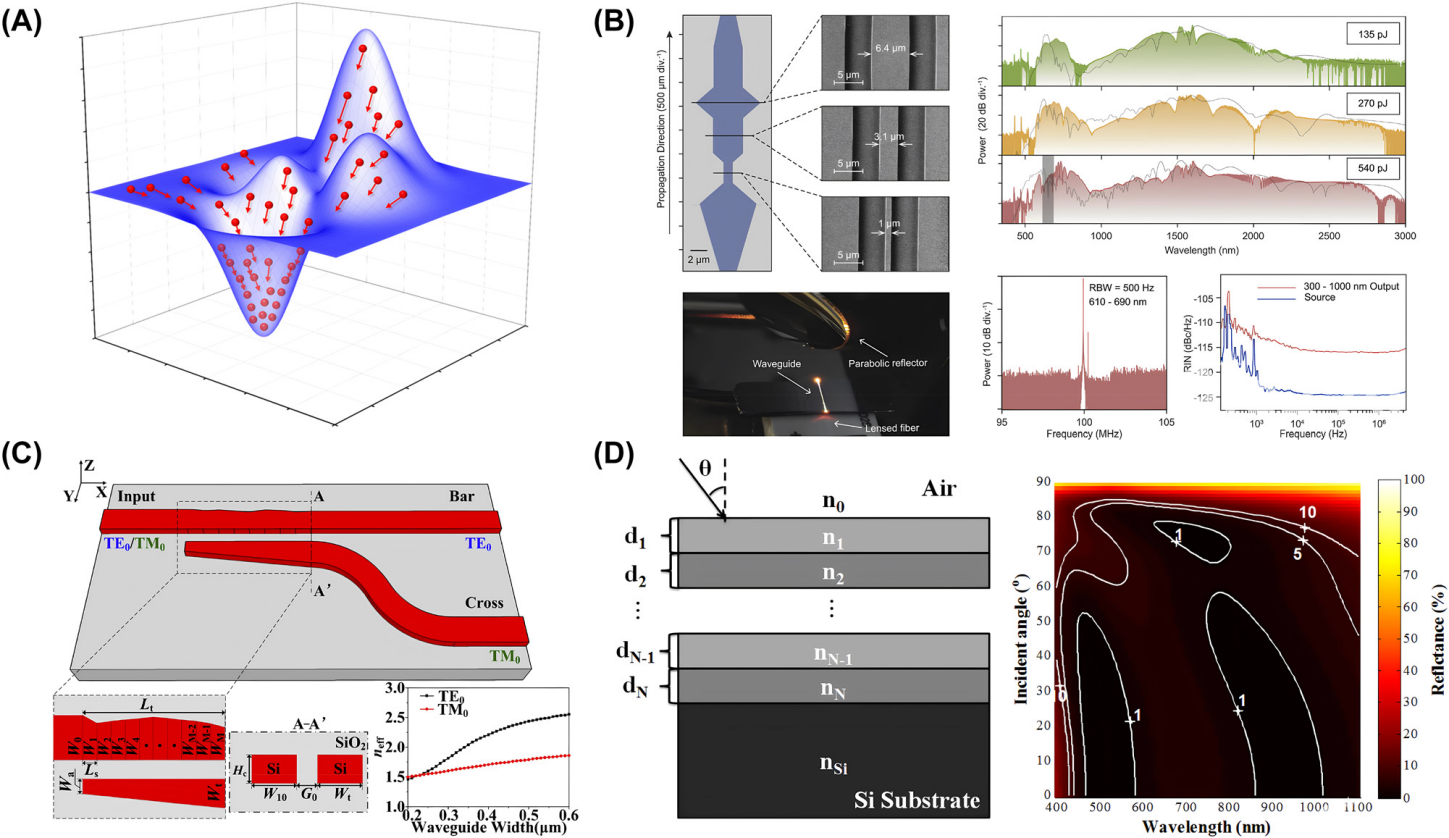
Representative examples of inverse-designed nanophotonic devices enabled by swarm-based algorithms. (A) Schematic of searching mechanism in particle swarm optimization. (B) Experimental characterization of inverse-designed freeform waveguides enabled by particle swarm optimization. (C) Inverse-designed silicon polarizer beam splitter using particle swarm optimization. (D) Inverse-designed omnidirectional antireflection coatings using ant colony optimization method. (B) Is reprinted from Ref. [34], with permission (CC BY 4.0); (C) is reprinted from Ref. [35], under the terms of the Open Access Publishing Agreement; (D) is reprinted from Ref. [36], under the terms of the Open Access Publishing Agreement.

Several inverse-designed nanophotonic devices leveraging swarm-based algorithms are shown in Figure 3. These recent results emphasize the potential of these methods for designing various nanophotonic devices, particularly for tasks involving relatively continuous design parameters.

### 2.2 Adjoint methods

In sharp contrast to the aforementioned heuristic optimization techniques commonly used in photonics, a more direct and intuitive approach to finding the optimal point is to define an objective quantity as a function of optical structures (i.e. geometry and material properties) that is to be minimized. Then, by deriving the gradient of the objective function with respect to the structure, we can move toward the optimal point with the desired optical response along the steepest descent direction.

The challenge, however, lies in the vast number of parameters that constitute the design space, making it computationally expensive to calculate each element of the gradient one by one. This approach requires at least *N* + 1 full-wave simulations for *N*-dimensional parameters to approximate the first-order derivatives of the objective function in a brute-force manner. In this approach, solving *N* linear equations of the form *y*_*n*_ – *y*_0_ = **∇** *L*(**x**_0_) · (**x**_*n*_ – **x**_0_) for *n* = 1, …, *N* derives the gradient **∇** *L*(**x**_0_) at a point **x**_0_, where *y*_*n*_ = *L*(**x**_*n*_) represent the objective function evaluated at *N* additional points in the vicinity of **x**_0_. Adjoint-sensitivity optimization, on the other hand, offers an elegant solution to this complexity by leveraging the chain rule in the mathematical expression of the gradient, reducing the required computations to just two simulations: one forward and one adjoint (backward).

Following the description in Ref. [37], a simplified optimization problem is considered: *ϕ*_opt_ = argmin *L*(e, e^*^; *ϕ*) such that *f*(e, e^*^, *ϕ*) = 0, where *ϕ* is a parameter vector, *L* is the objective function to be minimized, and e and e^*^ are the complex state vector and its corresponding complex conjugate, both implicitly functions of *ϕ*. The function *f* represents the multiple constraints (i.e. the vectorial governing equation) which typically have the same dimension as e. The expression for the total derivative of *L* with respect to *ϕ* is given by

(5) $$\begin{array}{ll} \frac{\text{d}L}{\text{d}\phi } & =\frac{\partial L}{\partial \phi }+\frac{\partial L}{\partial \text{e}}\frac{\partial \text{e}}{\partial \phi }+\frac{\partial L}{\partial {\text{e}}^{*}}\frac{\partial {\text{e}}^{*}}{\partial \phi }\\ & =\partial_{\phi }L+\left[\begin{array}{cc} \partial_{\text{e}}L & \partial_{{\text{e}}^{*}}L \end{array}\right]\left[\begin{array}{c} \partial_{\phi }\text{e}\\ \partial_{\phi }{\text{e}}^{*} \end{array}\right], \end{array}$$

where ∂_*ϕ*_ *L*, ∂_e_ *L* and ∂_e*_ *L* are straightforward to derive from the explicit dependence of *L*, while ∂_*ϕ*_e and ∂_*ϕ*_e^*^ are only accessible through solving the governing equation. Here, the chain rule is applied similarly to *f* and *f* ^*^, which can be written in matrix form as:

(6) $$\left[\begin{array}{c} \partial_{\phi }\text{f}\\ \partial_{\phi }{\text{f}}^{*} \end{array}\right]=-\left[\begin{array}{cc} \partial_{\text{e}}\text{f} & \partial_{{\text{e}}^{*}}\text{f}\\ \partial_{\text{e}}{\text{f}}^{*} & \partial_{{\text{e}}^{*}}{\text{f}}^{*} \end{array}\right]\left[\begin{array}{c} \partial_{\phi }\text{e}\\ \partial_{\phi }{\text{e}}^{*} \end{array}\right].$$

Substituting the relationship in Eqs. (6) into (5), the total derivative becomes:

(7) $$\begin{array}{ll} \frac{\text{d}L}{\text{d}\phi } & =\partial_{\phi }L-\left[\begin{array}{cc} \partial_{\text{e}}L & \partial_{{\text{e}}^{*}}L \end{array}\right]{\left[\begin{array}{cc} \partial_{\text{e}}\text{f} & \partial_{{\text{e}}^{*}}\text{f}\\ \partial_{\text{e}}{\text{f}}^{*} & \partial_{{\text{e}}^{*}}{\text{f}}^{*} \end{array}\right]}^{-1}\left[\begin{array}{c} \partial_{\phi }\text{f}\\ \partial_{\phi }{\text{f}}^{*} \end{array}\right]\\ & =\partial_{\phi }L+2\text{Re}\left({\text{e}}_{\text{adj}}^{T}\cdot \partial_{\phi }\text{f}\right), \end{array}$$

where e_adj_ and ${\text{e}}_{\text{adj}}^{*}$ define the adjoint fields, satisfying the adjoint simulation:

(8) $$\left[\begin{array}{cc} {\left(\partial_{\text{e}}\text{f}\right)}^{T} & {\left(\partial_{\text{e}}{\text{f}}^{*}\right)}^{T}\\ {\left(\partial_{{\text{e}}^{*}}\text{f}\right)}^{T} & {\left(\partial_{{\text{e}}^{*}}{\text{f}}^{*}\right)}^{T} \end{array}\right]\left[\begin{array}{c} {\text{e}}_{\text{adj}}\\ {\text{e}}_{\text{adj}}^{*} \end{array}\right]=-\left[\begin{array}{c} {\left(\partial_{\text{e}}L\right)}^{T}\\ {\left(\partial_{{\text{e}}^{*}}L\right)}^{T} \end{array}\right],$$

with adjoint sources ∂_**e**_ *L* and $\partial_{e^{*}}L$ derivable from the forward simulation.

**Figure 4:**
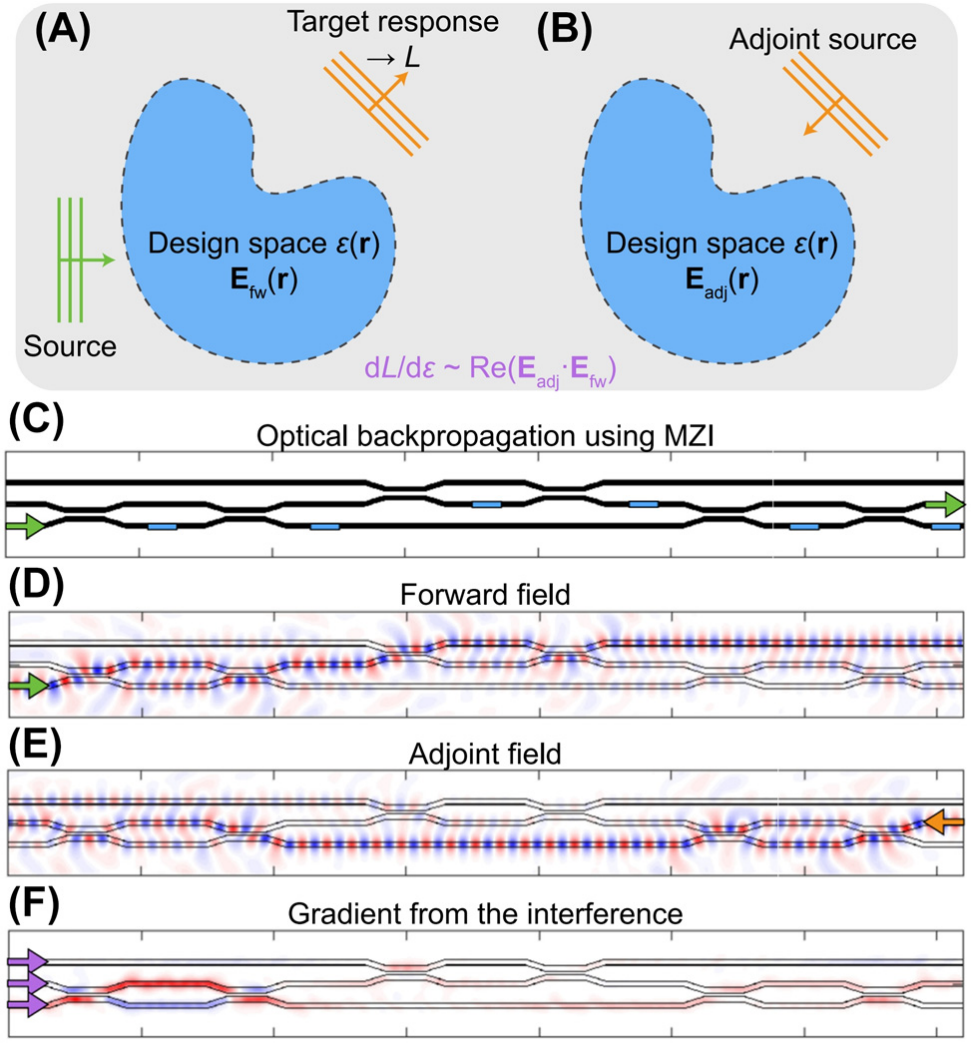
Concept of adjoint-sensitivity optimization. (A) Forward simulation involves calculating the objective function (*L*) by comparing the simulated field (**E**_fw_) with the target response. (B) The adjoint simulation uses a backward source derived from *L* to generate the adjoint field (**E**_adj_). The gradient of *L* with respect to the permittivity distribution *ε*(**r**) within the design space is then derived as the product of forward and adjoint fields. (C–F) Optical gradient backpropagation through the adjoint method in analogy with that of deep neural network. Green arrows, forward input; blue area and rectangles, design region; orange arrows, adjoin sources; purple arrows, forward and time-reversed adjoint sources combined to interfere with two fields. Panels (C–F) are adapted with permission from Ref. [42].

In general photonics problems with isotropic, nonmagnetic and monochromatic assumptions, the wave equation is expressed as [∇^2^ + *k*_0_^2^ *ε*(*r*)]*E*(*r*) – *j*(*r*) = 0, where *k*_0_ = *ω*/*c* is the free-space wave number, *ε* is the relative permittivity distribution, and *E* is the electric field, and *j* represents current density distribution as a forward source (as shown in Figure 4A). By setting *E*(*r*) and *ε*(*r*_0_) as e and scalar *ϕ* in Eqs. (7) and (8), ∂_e_ *f* corresponds to the operator inside the bracket applied to *E*(*r*), which happens to be a symmetric operator. Consequently, (∂_e_ *f*)^*T*^ = ∂_e_ *f*, meaning that solving Eq. (8) is equivalent to solving the standard wave equation with an adjoint source *j*_adj_ = –(∂_e_ *L*)^*T*^ (Figure 4B). The resulting electric field *E*_adj_(*r*) gives rise to the single dimensional derivative when multiplied by ∂_*ϕ*_ *f* := *δ*(*r* – *r*_0_)*E*_fw_(*r*). This approach can be easily extended to multi-dimensional vector *ϕ*, still requiring only two (forward and adjoint) simulations. It is also worth noting that this formulation can be readily modified to handle the generalized wave equation, including time-dependence [38], [39], nonlinearity [37], [40], and/or nonreciprocity [41].

Interestingly, the process of gradient calculation via adjoint simulation directly parallels the concept of “backpropagation” in deep neural networks, both in their chain-rule-based formulation and in the forward-then-backward flow of information. Based on this tight analogy between two concepts from different domains, Hughes et al. [42] demonstrated the *in-situ* implementation of backpropagation in an optical neural network as shown in Figure 4C–F. Figure 4C displays a waveguide mesh structure based on Mach–Zehnder interferometers (MZIs) with phase shifters (blue area) [43], capable of performing an arbitrary 3-by-3 unitary operation between input (|3〉) and output (|2〉) vectors (green arrows). To obtain gradient information within the blue areas for the target operation |2〉〈3|, one needs to (1) inject the forward field as shown in Figure 4D and store the intensity, *I*_fw_ = |*E*_fw_|^2^ within the blue areas, (2) inject the adjoint field as shown in Figure 4E and store the intensity *I*_adj_ = |*E*_adj_|^2^, (3) inject the forward and time-reversed adjoint field again at the front end as shown in Figure 4F and record the interference pattern, *I*_interf_ = |*E*_fw_ + *E*_adj_^*^|^2^ = *I*_fw_ + *I*_adj_ + 2Re(*E*_adj_ *E*_fw_). Through this procedure, the gradient term Re(*E*_adj_ *E*_fw_) can be obtained *in-situ* as *I*_interf_ – *I*_fw_ – *I*_adj_. This example bridges the adjoint method and deep neural networks, enabling not only inference but also *in-situ* training of optical neural networks exactly in the same manner as traditional deep neural networks.

**Figure 5:**
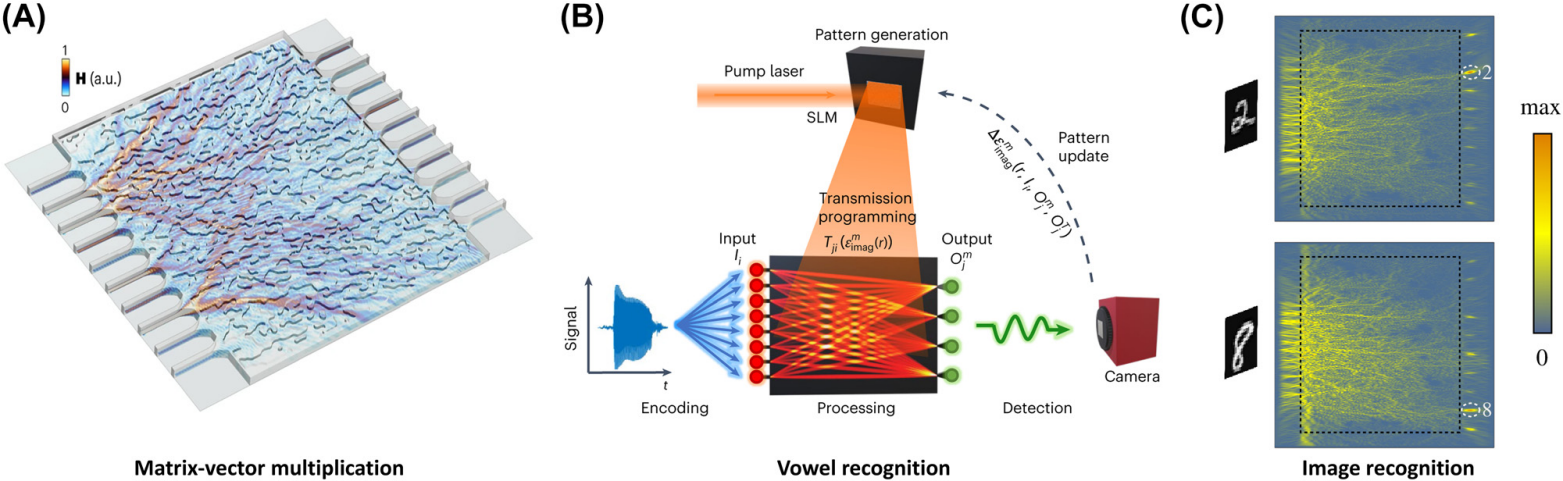
Optical computers designed with the adjoint method. (A) Silicon photonics platform for matrix-vector multiplication with a very high dimensionality (10 × 10). (B) Lithography-free trainable processor for vowel recognition. (C) Neuromorphic continuous medium including nonlinearity for image recognition. (A) Is reproduced from Ref. [44], with permission from SNCSC; (B) is reproduced from Ref. [45], with permission from SNCSC; (C) is reprinted from Ref. [40], with permission from Photonics Research.

Among its various applications, this shift towards intelligence-driven optimization techniques has significantly advanced photonic devices as a computing platform, particularly in large-scale, reconfigurable, and neuromorphic applications. Here, we present a few examples where the adjoint method was utilized in the design of optical computing platforms. First, Figure 5A showcases the optical matrix-vector multiplication [44], as described in Ref. [42], but using a free-form Si core with binary thicknesses (220 and 150 nm) immersed in an SiO_2_ cladding, operating at 1,525 nm wavelength. Due to the large footprint of the device, approximately 30 μm, the 2D effective index approximation technique was employed in the design of the 3D planar structure to reduce the computational cost. This combined approach, utilizing the adjoint-sensitivity method and theoretical effective medium approximation, significantly enhances scalability, which is crucial for practical use as a linear layer in deep neural networks.

Next, Figure 5B demonstrates the *in-situ* training of an optical gain medium for vowel recognition [45]. Unlike the phase shifters used in the MZI structure [42], this approach utilizes a spatially modulated pump beam to control the gain parameters across the design area, enabling targeted neuromorphic functionality. Figure 5C further demonstrates a nanophotonic medium that incorporates a nonlinear component, functioning as a nonlinear activation function in a deep neural network. This configuration is used for recognizing handwritten images, routing the flow of light into different paths based on the visual information contained in the input light.

### 2.3 AI-enabled design approaches

As discussed in the previous section, optimization algorithms have been well-established over the past few decades, resulting in superior designs and performances. However, these algorithms are often constrained by high computational costs, which can render the design process inefficient. Artificial intelligence (AI) has the capability to immediately map structural information to its corresponding optical responses, potentially replacing the time-consuming electromagnetic simulation. Here, we need only one-time cost simulations to train the AI by extensively running electromagnetic simulations that explore a sufficiently large portion of the parameter space for a specific type of device with standardized parameter space. Once trained, the model is capable of predicting the optical responses in microsecond order and can be applied to a range of related design problems. Over the last decade, numerous efforts have been made to implement this novel technique to design nanophotonic devices [46], [47]. There are several ways to inverse-design nanophotonic devices with AI and in this section, these methods are classified into three categories: discriminative models, generative models, and reinforcement learning.

#### 2.3.1 Inverse design with discriminative models

**Figure 6:**
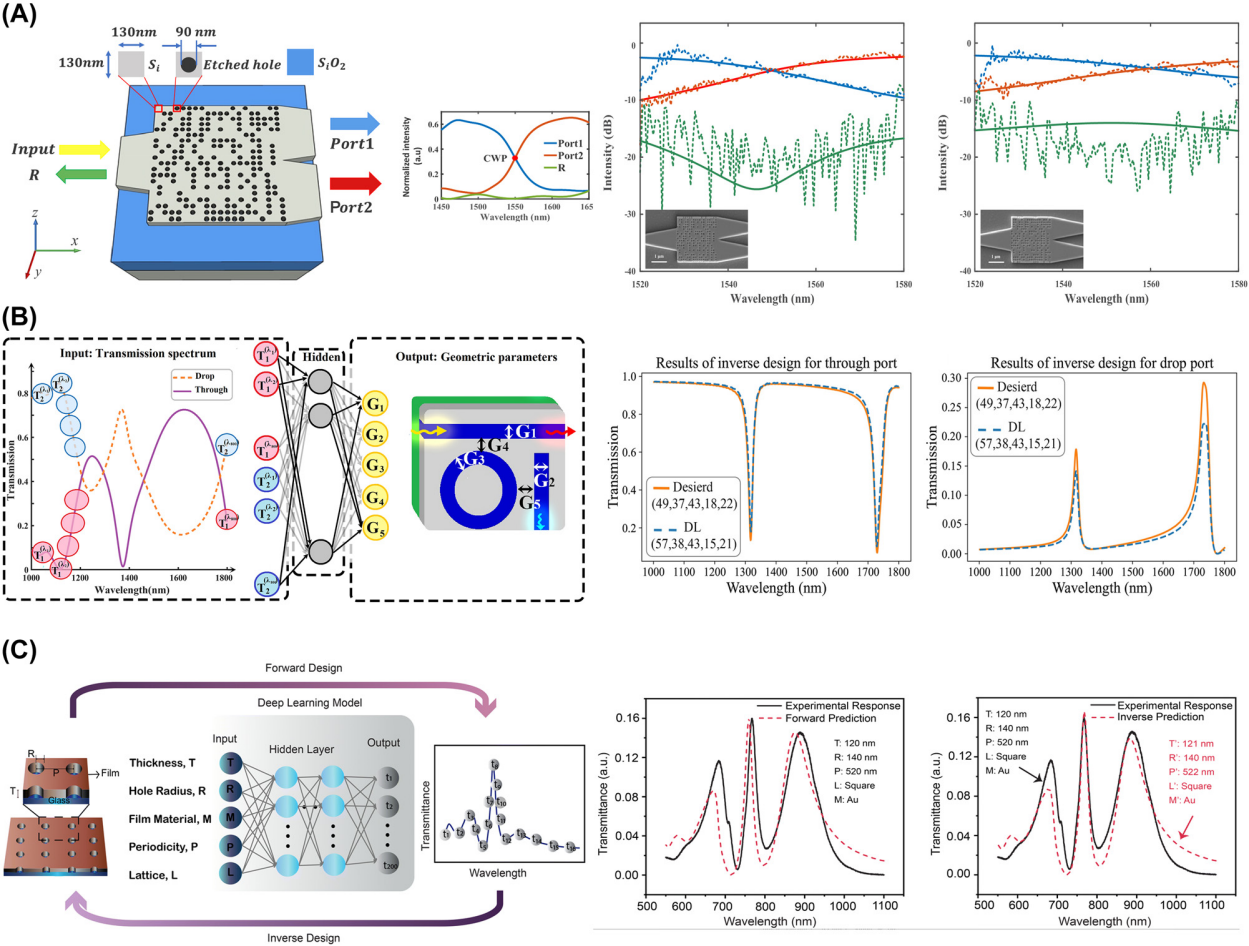
Representative examples of inverse-designed nanophotonic devices enabled by discriminative models. (A) Schematic of the inverse-designed 1 × 2 wavelength demultiplexer (left) and experimental verification (right). (B) Schematic of the all-optical nonlinear plasmonic ring resonator switch and the structure of the inverse design network (left), and training results (right). (C) Inverse design configuration of nanophotonic nanohole arrays (left), and experimental verification (right). (A) Is reprinted from Ref. [48], under the terms of the Open Access Publishing Agreement; (B) is reprinted from Ref. [49], with permission (CC BY 4.0); (C) is reprinted from Ref. [50], with permission. Copyright 2024 Royal Society of Chemistry.

Discriminative models have been extensively applied across various fields, including computer vision, image processing, and natural language processing, where the primary goal is to learn a direct relationship between input data and output labels. In nanophotonic design, discriminative models, such as fully connected neural networks (FCNs) and convolutional neural networks (CNNs), play a critical role in both forward and inverse design tasks. As described in Figure 6, these models allow for the mapping of structure parameters, such as the geometric details of nanostructures, to their corresponding optical responses, such as reflection or transmission spectra, or inversely, to predict the structural parameters required to achieve a desired optical outcome.

FCNs, one of the simplest forms of discriminative models, consist of layers of interconnected neurons where each neuron is connected to every neuron in adjacent layers. FCNs are particularly useful for capturing relationships between device structural parameters, such as material properties, thickness, or periodic patterns and their corresponding optical responses. For example, an FCN can predict the spectral response of a nanophotonic device based on a given set of design inputs as demonstrated in Ref. [24]. While FCNs are effective for simpler design tasks involving basic geometries, they often encounter difficulties when handling high-dimensional data and more complex design problems, as the increased complexity of the input data leads to an exponential growth in parameters. This results in the need for substantial training data and prolonged training periods, thereby restricting their effectiveness for more sophisticated designs.

To address these limitations, CNNs are often employed for more complex inverse design problems in nanophotonics. Unlike FCNs, CNNs are designed to handle high-dimensional data such as images or complex patterns, making them well-suited for processing intricate geometric features in nanophotonic design [21]. Through convolutional layers, CNNs can extract local features from the input data, enabling the model to capture spatial hierarchies and relationships within the structure. This is particularly powerful in nanophotonic design, where a device’s optical properties are highly sensitive to its detailed geometric configuration.

Despite these advantages, discriminative models in inverse design face challenges, most notably the “one-to-many mapping” problem. This issue arises when different structural configurations produce the same optical response, complicating the task of predicting a unique set of design parameters. To address this, advanced techniques such as tandem networks and mixture density networks (MDNs) have been proposed. Tandem networks involve training a forward model to map design parameters to optical responses and then using this pre-trained model to guide an inverse network in predicting the specific structural parameters that match a given optical outcome. This approach enables one-to-one mapping, thereby enhancing the accuracy and reliability of the inverse design process. MDNs offer another solution by employing a probabilistic approach to capture the non-uniqueness problem in inverse design. Instead of providing a single predicted outcome, MDNs generate a distribution of possible design solutions, allowing the exploration of multiple configurations that achieve the desired optical outcomes. This is particularly advantageous for exploring complex design spaces where numerous valid solutions exist.

Alongside these advanced techniques like tandem networks or MDNs, the primary strength of discriminative models lies in their ability to provide rapid and precise predictions, significantly reducing the computational time and resources, compared to the optimization algorithm-based inverse design method. However, they are constrained by their reliance on existing data, which limits their ability to explore new design spaces. This limitation has led to growing demand for solutions that can uncover new design possibilities and explore more sophisticated and unexplored areas, a challenge that generative models are particularly well-equipped to address.

#### 2.3.2 Inverse design with generative models

Generative models have recently achieved significant breakthroughs across various domains such as image processing, large language models (LLMs), natural language processing (NLP), computer vision (CV), and the automotive industry. Notable examples that have implemented generative models include DALL-E 2 and ChatGPT, both of which have demonstrated superior performance. This growing interest has accelerated further advancements in generative models, encompassing techniques such as variational autoencoders, generative adversarial networks, diffusion models, and transformers. Following their successful applications in image, video, and language processing, researchers are now expanding the use of generative models to additional fields, including photonics, material science, healthcare, protein folding, environmental science, and beyond.

Variational autoencoders (VAEs) are well-established generative models that employ an encoder structure to extract a latent space capturing meaningful information [51]. Outputs are then generated using the decoder structure that reconstructs data from this latent space. During training, a VAE updates the parameters of both the encoder and decoder, enabling it to generate new data. To enhance performance, variations of the VAE, such as the conditional VAE (CVAE), are widely applied.

Generative adversarial networks (GANs) are also well-known generative models [52]. Unlike VAEs, GANs consist of two distinct networks: the generator network and the discriminator network. The purpose of the generator network is to create a synthetic distribution that closely resembles the real distribution.

Conversely, the purpose of the discriminator network is to differentiate between the generated and real distributions. During the training process, these two networks engage in a competitive process, where the discriminator’s probability of distinguishing between real and fake data approaches approximately 0.5. This outcome indicates that the discriminator can no longer effectively differentiate between the generated data and real data. Several different versions of GAN have also been developed, such as deep convolutional GAN (DCGAN) or conditional DCGAN (cDCGAN).

The diffusion model is a recently emerged generative network, which outperforms VAEs and GANs [53]. It first transforms the original image into a complex data distribution by gradually adding a simple noise distribution, a process called diffusion. The network is then trained to denoise this complex data distribution step-by-step, moving backward through the time steps. During the training process, the network learns to predict the added noise at each step, enabling it to denoise the complex sample while minimizing the difference between the true noise and the predicted noise.

**Figure 7:**
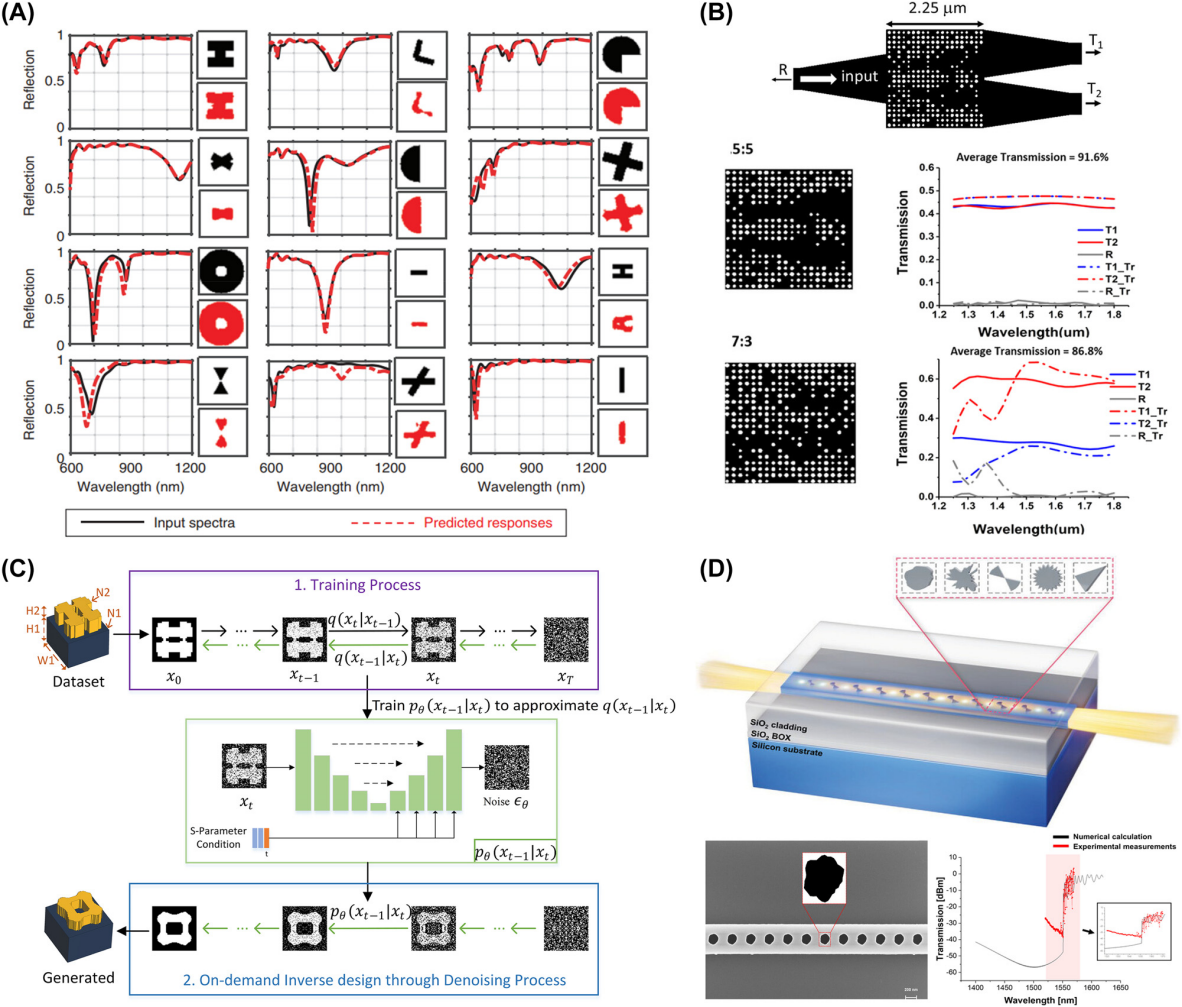
Representative examples of inverse-designed nanophotonic devices enabled by generative models. (A) Twelve examples of inverse-designed unit cells in metasurfaces and their optical characteristics suggested by cDCGAN. (B) Schematic of the inverse-designed 1 × 2 photonic power splitter by leveraging CVAE (upper) and simulation results with arbitrary splitting ratios (down). (C) Schematic of the diffusion model-enabled inverse design process. High-degree-of-freedom metasurfaces can be inverse designed, successfully mimicking the original shape. (D) Schematic of the inverse-designed nanophotonic device with deep generative models (left) and SEM image of the fabricated device with experimental result. (A) Is reprinted from Ref. [54], with permission (CC BY 4.0); (B) is reprinted from Ref. [55], with permission. Copyright 2024 John Wiley and Sons; (C) is reprinted from Ref. [56], with permission (CC BY 4.0); (D) is reprinted from Ref. [57], with permission. Copyright 2024 John Wiley and Sons.

In the field of nanophotonics, generative models are utilized to generate new structures that are not present in the original dataset but successfully replicate the interesting optical properties (see Figure 7). These models can replace the highly time-consuming design process once a sufficient number of designs are available.

#### 2.3.3 Inverse design with reinforcement learning

Reinforcement learning (RL) is a different type of AI technique, where an agent learns to make decisions by receiving feedback from its environment [58]. Following the success of RL in several tasks including Go, chess, and video games, it has received significant attention across various fields [59], [60], [61], [62]. By leveraging RL to explore complex design spaces, abundant nanophotonic devices can be optimized, including waveguides, metasurfaces, gratings, and beyond. During the training process, the agent optimizes the structure by evaluating optical properties, such as efficiency, bandwidth, and wavelength selectivity.

**Figure 8:**
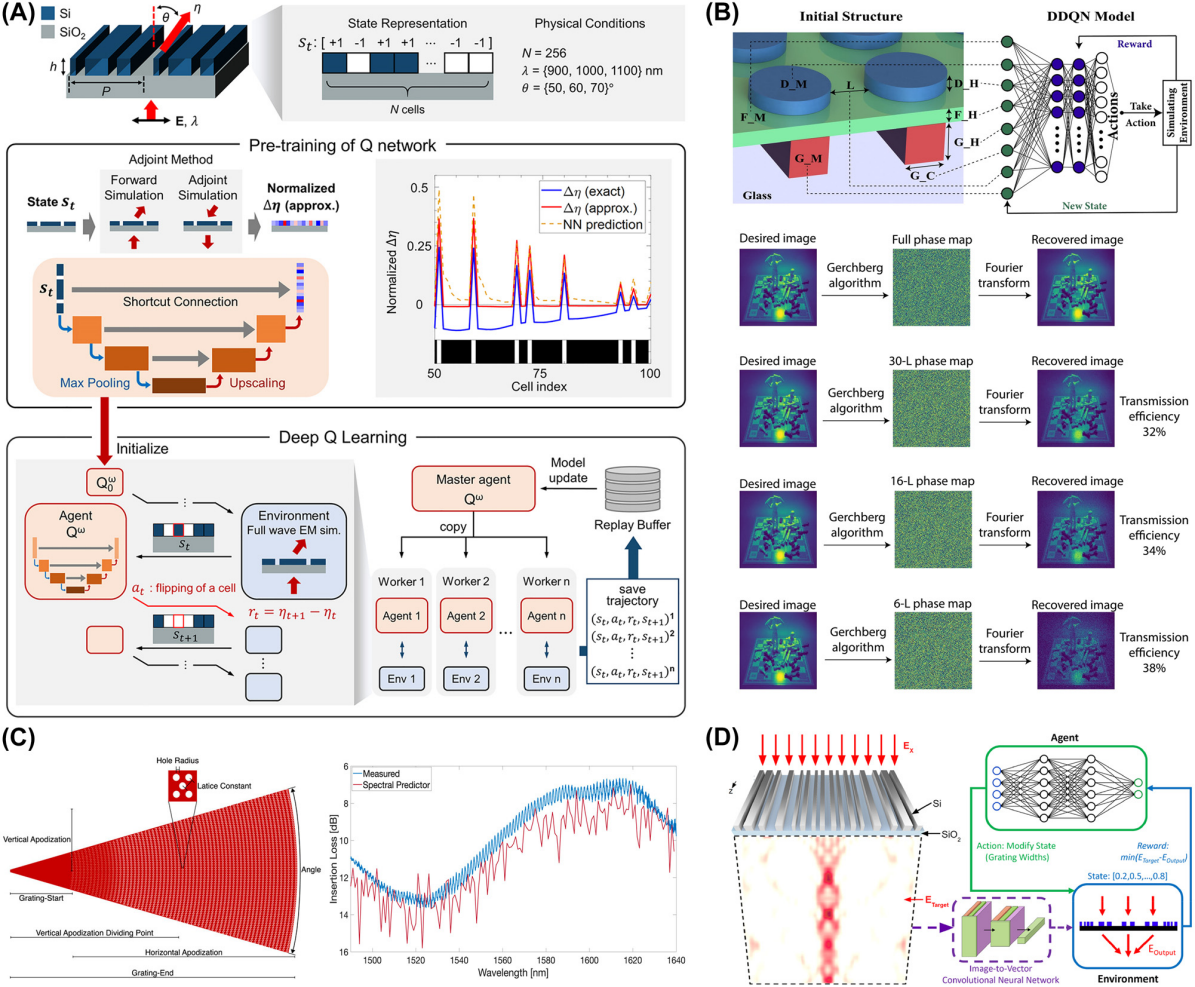
Representative examples of inverse-designed nanophotonic devices enabled by reinforcement learning. (A) An overview of physics-informed RL consists of the pre-training stage and RL optimization stage. (B) The schematic of DDQN-based design of metasurfaces and simulation results of high-quality metasurface holograms. (C) The schematic of the parameterized grating coupler inverse-designed with RL, microscopic image of fabricated devices, and experimental verifications. (D) Inverse design of metagrating structure leveraging RL combined with the supervised learning method. (A) Is reprinted from Ref. [63], with permission (CC BY 4.0); (B) is reprinted from Ref. [64], with permission (CC BY 4.0); (C) is reprinted from Ref. [65], with permission (CC BY 4.0). (D) Is reprinted from Ref. [66], under the terms of the Open Access Publishing Agreement.

Implementing RL in nanophotonics involves several key steps. First, an environment is created in which the RL agent will be trained. This can include design parameters of the device itself (e.g. period, radius, width, height) or optical properties (e.g. refractive index distribution, polarization, phase). After defining the environment, the reward process is defined. Based on the RL target task, the agent needs to define the amount of reward to determine the next action. The reward may be based on performance metrics related to optical characteristics such as maximizing efficiency, enlarging bandwidth, and maximizing transmission. Note that these optical characteristics are usually obtained through numerical calculations such as the finite-difference time-domain (FDTD) method, finite element method (FEM), and others. Next, the agent is defined, and it begins taking actions to update the environment. Finally, the agent is trained. Starting from the initial state of the nanophotonic structure, the reward is numerically calculated and the agent takes actions to update the structural information. RL repeats this process to inversely design nanophotonic devices as shown in Figure 8. Several methods can be utilized for RL, including deep Q-learning (DQN), double DQN (DDQN), and others.

## 3 Inverse-designed nanophotonic devices: selected examples

Inverse design has revolutionized the field of nanophotonics, facilitating the optimization of photonic devices through metaheuristic algorithms, adjoint methods, and AI-driven designs discussed in previous chapter. This approach has led to the creation of next-generation devices, including power splitters, wavelength (de)multiplexers, grating couplers, and waveguide devices. Metasurfaces have also seen significant improvements, achieving new performance levels in light control. Beyond nanophotonics, inverse design is also expanding into material science and mechanical engineering, proving its potential across diverse domains. This chapter will explore these advancements and highlight how inverse design is driving the evolution of photonics and other areas of research.

### 3.1 Nanophotonic power splitters

**Figure 9:**
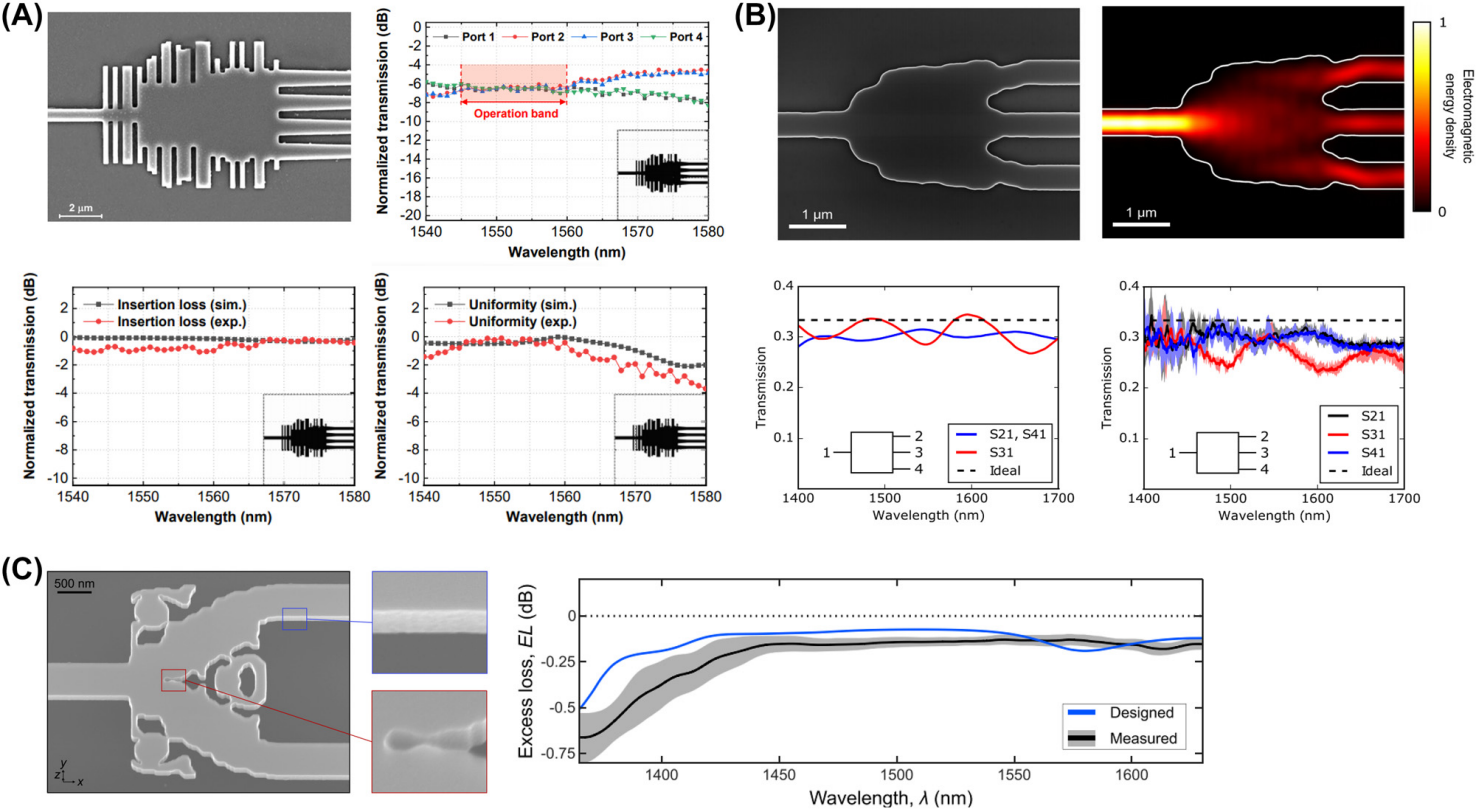
Representative examples of inverse-designed nanophotonic power splitters. (A) Inverse-designed 1 × 4 nanophotonic power splitter using PSO. The fabricated device’s scanning electron microscopy (SEM) image (upper left) and measured transmission spectrum of the 1 × 4 power splitter (upper right). Comparison between the simulated and measured spectra for the insertion loss (lower left) and comparison between the simulated spectra and measured spectra for the uniformity loss (lower right). (B) Inverse-designed 1 × 3 nanophotonic power splitter with TO. SEM image of the fabricated device (upper left) and simulated field propagation showing light distribution into three ports (upper right). Simulated spectra of the transmission for each port (lower left) and measured spectra of the transmission for each port (lower right). (C) Inverse-designed 1 × 2 nanophotonic power splitter with TO. SEM image of the fabricated device (left) and comparison of the simulated and measured transmission spectra (right). (A) Is reprinted from Ref. [69], with permission (CC BY 4.0); (B) is reprinted from Ref. [70], with permission (CC BY 4.0); (C) is reprinted from Ref. [71], with permission (CC BY 4.0).

Various research results have been implemented for nanophotonic power splitters, employing methods ranging from conventional heuristic algorithms like PSO or DBS to advanced topology optimization (TO) as demonstrated in Figure 9. Many of these have been experimentally verified and some studies have explored the potential for designing splitters using deep learning techniques. By applying inverse design methods, nanophotonic power splitters can be engineered with significantly smaller footprints than conventional structures, such as those utilizing directional coupler (DC) [67] or multimode interference (MMI) types [68]. These inverse-designed splitters can distribute light into multiple output ports, such as 1 × 3, 1 × 4, or 1 × 8, rather than being limited to 1 × 2, allowing for the desired distribution ratios and minimal optical loss across a broadband range.

For instance, Kim et al. detailed the optimization of a 1 × 4 splitter using the PSO algorithm. Two different devices were fabricated, each composed of 40 rectangular shapes with varying widths, designed to optimize the arrangement of 40 particles [69]. The dimensions of the devices were compact, measuring 6.0 × 7.2 μm^2^ and 8.4 × 12 μm^2^, respectively. The outcomes demonstrated a maximum insertion loss of 0.76 dB and a uniformity of less than 0.84 dB for one device, and 1.08 dB and 0.81 dB for the other, closely matching the predicted simulation results. Similarly, Xie et al. described the use of the DBS algorithm to simulate and experimentally validate a 1 × 4 power splitter capable of splitting in ratios such as 1:1:1:1, 2:2:1:1, 2:2:2:1, and 4:3:2:1 over a 2 μm wavelength band, achieving a compact size of 3.6 × 3.6 μm^2^ [72].

Following these heuristic approaches, advanced TO methods were also applied to enhance the design and performance of power splitters. Piggott et al. demonstrated a 1 × 3 splitter using TO with curvature constraints to prevent small feature formations, setting a minimum curvature radius of 100 nm. The fabricated device showed an insertion loss of 0.642 ± 0.057 dB and power uniformity of 0.641 ± 0.054 dB across a 1,400–1,700 nm wavelength range [70]. Xu et al. applied digital TO, enhancing traditional methods by incorporating process-oriented constraints such as minimum feature size and edge smoothing [73]. This led to the successful simulation and optimization of a broadband 1 × 2 splitter that supports TE0/TE1 mode beam splitting over a wavelength bandwidth of 445 nm and a compact size of 5.4 × 2.88 μm^2^. Hansen et al. further demonstrated the experimental validation of a TO-optimized 1 × 2 power splitter, achieving an impressively low excess loss of under 0.5 dB across a 245 nm wavelength range [71].

Table 1 presents additional results on power splitters using inverse design methods, as reported in various papers.

**Table 1:** Summary of inverse-designed power splitters.

Ref	Design method	Structure	IL (dB) (sim./exp.)	UL (dB) (sim./exp.)	BW (nm) (sim./exp.)	Foot print (μm^2^)
[67]	Forward (DC)	1 × 2	–/1	–/0.7	100/88	31.4 × 1.3
[68]	Forward (MMI)	1 × 2	0.3/0.3	–/0.6	200/171	43 × 3.1
[71]	TO	1 × 2	0.1/0.1	–	325/245	2 × 3
[73]	TO	1 × 2	0.8/–	–	447/–	5.4 × 2.9
[70]	TO	1 × 3	0.8/0.6	0.5/0.6	300/300	3.8 × 2.5
[74]	DBS	1 × 3	1.9/–	N/A	100/–	77.2
[75]	DL	1 × 3	0.45/–	N/A	200/–	2.6 × 2.6
[72]	DBS	1 × 4	1/1.5	N/A	40/30	3.6 × 3.6
[76]	PSO	1 × 4	–/0.6	–/1.0	–/44	12.3 × 5
[77]	PSO	1 × 4	0.6/0.6	0.3/0.9	150/104	36 × 6
[77]	PSO	1 × 8	0.6/0.6	0.8/0.8	150/104	47.8 × 11.3

### 3.2 Wavelength (de)multiplexers

**Figure 10:**
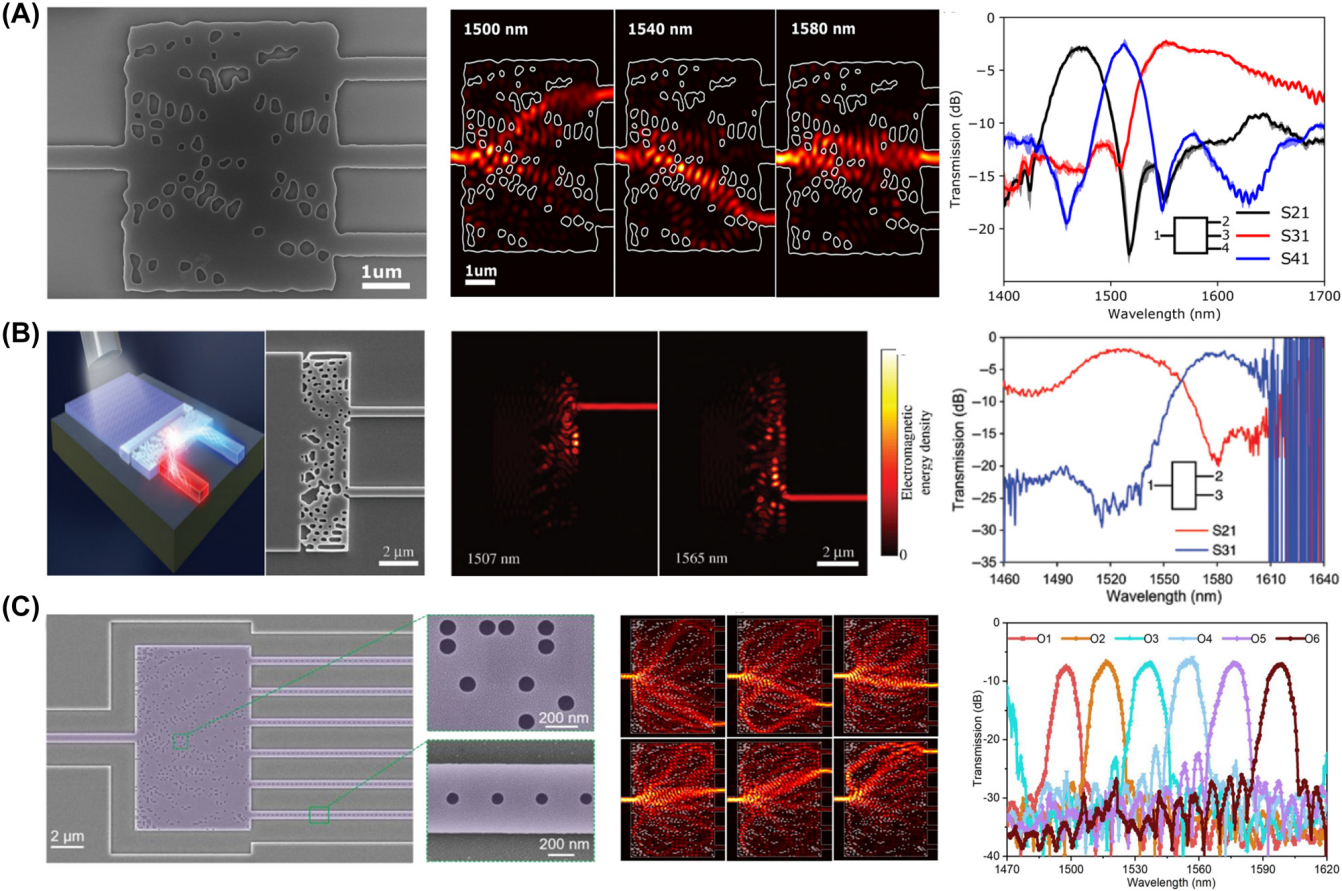
Representative inverse-designed nanophotonic wavelength (de)multiplexers. (A) Inverse-designed 1 × 3 wavelength demultiplexer with TO. SEM image of the fabricated device (left), simulated field propagation results for the optimized structure, varying the wavelength from 1,500 nm to 1,580 nm in 40 nm increments (middle) and measured transmission spectra of the device for each port (right). (B) Inverse-designed 1 × 2 wavelength demultiplexer with TO. Whole scheme and SEM image of the fabricated device (left), simulated field propagation results for the optimized structure, varying the wavelength at 1,507 nm and 1,565 nm (middle) and measured transmission spectra for each port (right). (C) Inverse-designed 1 × 6 nanophotonic power splitter optimized with DBS algorithm and additional photonic crystal structures. SEM image of the fabricated device with a magnified view of the nanoholes and PhC structure (left), simulated field propagation results for the optimized structure, varying the wavelength from 1,500 nm to 1,600 nm in 20 nm increments (middle) and measured transmission spectra for each port (right). (A) Is reprinted from Ref. [86], with permission. Copyright 2024 American Chemical Society; (B) is reprinted from Ref. [87], with permission (CC BY 4.0); (C) is reprinted from Ref. [88]. Copyright 2024 IEEE.

In conventional designs, wavelength (de)multiplexers have employed structures such as micro-rings [78], [79], subwavelength-grating (SWG)-based contra-directional coupler (contra-DC) filters [80], [81], cascaded Mach–Zehnder interferometers (MZI) structures [82], [83], or arrayed waveguide gratings (AWGs) [84], [85]. Recently, inverse design methods have been increasingly adopted for designing wavelength demultiplexers as shown in Figure 10, primarily employing TO or using DBS algorithms for pixelized design. These sophisticated approaches allow for enhanced performance and precise wavelength filtering within a compact device.

In 2015, Piggott et al. reported the design and fabrication of a 1 × 2 wavelength demultiplexer using TO, capable of splitting light from an input waveguide into two output waveguides at wavelengths of 1,300 nm and 1,550 nm [89]. The fabricated device achieved an insertion loss of less than 2.4 dB, crosstalk below −11 dB, and a 3-dB bandwidth exceeding 100 nm with notably compact dimensions of just 2.8 × 2.8 µm^2^. Su et al. incorporated self-biasing techniques into the TO process to enhance the robustness of the device against fabrication errors [86]. A 1 × 3 wavelength demultiplexer was designed and fabricated, capable of demultiplexing wavelengths of 1,500 nm, 1,540 nm, and 1,580 nm. The simulated performance showed an insertion loss of less than −1.55 dB and crosstalk levels below −15 dB. The fabricated device with a size of 5.5 × 4.5 µm^2^ demonstrated an insertion loss of less than −2.29 dB and crosstalk levels of −10.7 dB, slightly inferior to the simulation results. Huang et al. employed a refined TO approach to address the minimum feature size and performance degradation challenges [87]. The resulting device, with dimensions of 2.4 × 10 µm^2^, effectively demultiplexed wavelengths of 1,520 nm and 1,580 nm through a 10 µm-wide waveguide into two distinct ports. Experimental results confirmed insertion losses of −1.77 dB and −2.1 dB at each respective port, with crosstalk levels of −25.17 dB and −12.14 dB in the 1 × 2 wavelength multiplexer configuration.

Recent advancements have led to the development of structures using DBS algorithms with rectangular or nanohole-pixelized designs, which have increasingly become a leading choice for addressing the performance limitations of TO alone, particularly regarding insertion loss and crosstalk degradation post-fabrication. Recently, Deng et al. presented a hybrid analog-digital algorithm that begins with TO to determine the optimal structure, followed by an analog-to-digital conversion to create a pixelized structure, which is further refined using the DBS algorithm [90]. This method produced a device with compact size of 3 × 3 µm^2^, efficiently demultiplexed wavelengths at 1,550 nm and 2,000 nm, achieving insertion losses under 1.2 dB and 0.9 dB, with crosstalk levels below −17.7 dB and −16.4 dB. The device also demonstrated successful PAM-8 signal transmission at 138 and 84 Gbps for the respective wavelengths. Additionally, Wu et al. described an advanced device featuring an inverse-designed metastructure for wavelength demultiplexing, combined with cascaded photonic crystal filters to reduce crosstalk [88]. The design, optimized as a 1 × 6 demultiplexer using DBS algorithms, incorporated rectangular units with nanoholes. To further minimize crosstalk between ports, nanohole-based photonic crystal filters were added. The overall device dimensions were 27 × 12 µm^2^, with an insertion loss of 6 dB and crosstalk levels maintained below 20 dB. The results of these inverse-designed wavelength demultiplexer devices are summarized in Table 2.

**Table 2:** Summary of inverse-designed (de)multiplexers.

Ref	Design method	Structure	IL (dB) (sim./exp.)	XT (dB) (sim./exp.)	Footprint (μm^2^)
[79]	Forward (ring)	1 × 4	–/0.6	–/16	26 × 40 (single ring)
[80]	Forward (contra-DC)	1 × 4	–/1.8	–/−21.6	250 × 25 (single DC)
[82]	Forward (MZI)	1 × 4	–/3.7	–/−16	1,680 × 870
[89]	TO	1 × 2	2/2.4	−12.6/−11	2.8 × 2.8
[86]	TO	1 × 3	1.55/2.3	−15/−10.7	5.5 × 4.5
[87]	TO	1 × 2	1.45/2.1	−23.5/−12.1	2.4 × 10
[90]	TO + DBS	1 × 2	0.67/1.2	−19/−16.4	3.0 × 3.0
[88]	DBS	1 × 6	2.5/6	−38/−20	27 × 12

### 3.3 Grating couplers

**Figure 11:**
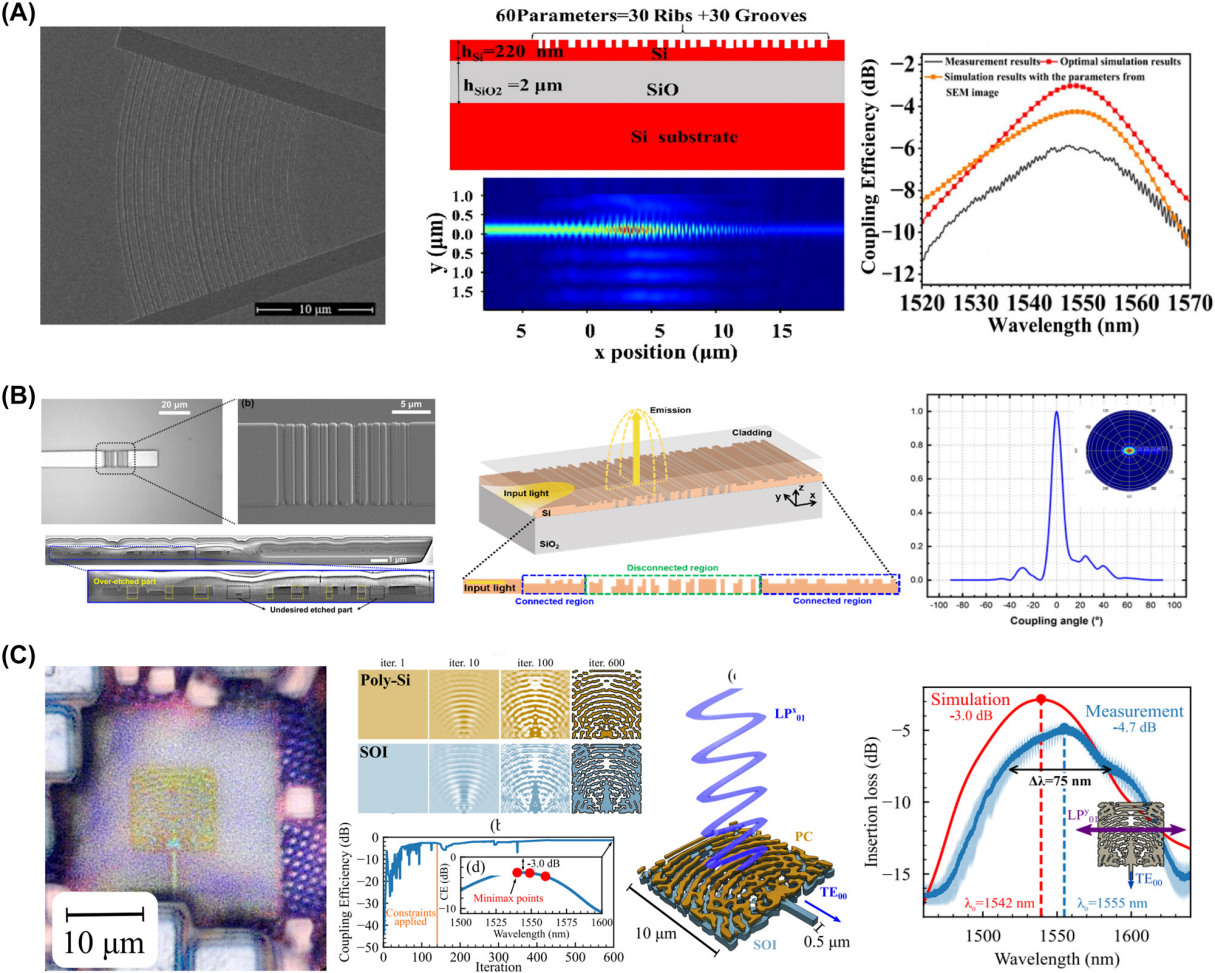
Representative inverse-designed grating couplers (GCs). (A) Gradient-based inverse-designed focused GC with a 70 nm etched structure. SEM image of the fabricated GC (left), gradient-optimized structure, and simulated field propagation (middle), and comparison between simulated coupling efficiency and measured coupling efficiency (right). (B) PSO-optimized meta GC with a triple-etched structure. SEM image of the fabricated device, including a magnified top and cross-sectional view (left), an illustration of the optimized structure (middle) and simulated radiation angle results showing vertical emission (right). (C) Multi-layer vertical emitting GC optimized by density-based TO. (a) Optical micrographs of the fabricated single-polarization GC (left). The density-based TO optimization process is depicted, showing the jointly optimized poly-Si and SOI layers, which achieved high coupling efficiency after 600 iterations (middle). A comparison between the simulated coupling efficiency and measured coupling efficiency (right). (A) Is reprinted from Ref. [98], with permission (CC BY 4.0); (B) is reprinted from Ref. [99], with permission from Photonics Research; (C) is reprinted from Ref. [100], under the terms of the Open Access Publishing Agreement.

In recent years, significant efforts have been dedicated to the designing grating couplers (GCs), essential components in optical I/O systems. Conventional straightforward approaches, such as chirped-grating [91], dual-etched grating [92], metallic mirror [93], and dual-layer grating designs [94], apodized grating [95], [96], [97] been proposed to enhance GC directivity and reduce back reflection. However, these methods often involve complicated fabrication processes that are incompatible with standard silicon photonics processes and have demonstrated limited performance. The inverse design approach has emerged as a promising alternative in designing GCs. While inverse-designed GCs do not achieve the dramatic size reductions seen in power splitters or wavelength multiplexers/demultiplexers, they offer greater design freedom. This enables GCs that better match the Gaussian beam profile at the intended coupling angle, thereby achieving higher coupling efficiency beyond what is achievable with conventional apodization techniques. Representative examples of inverse-designed GCs are depicted in Figure 11.

Yang et al. introduced an inverse-designed GC capable of fully vertical coupling [98]. This study utilized a gradient-based inverse design method implemented in the Python suite (Lumopt) to optimize a focused grating coupler with a 70 nm etch depth. The resulting device consisting of 30 periods, with rib and groove configurations was fabricated with a feature size of 100 nm. The measured coupling efficiency of the fabricated GC was −5.86 dB, representing a 3.04 dB improvement over conventional uniform GC. Nonetheless, there was a performance degradation of −2.86 dB compared to the simulation results, which was attributed to fabrication errors and difficulties in precise fiber alignment. Similarly, Sapra et al. applied a gradient-based inverse design method to design and fabricate out-of-plane GCs [101]. The GCs were optimized for target bandwidths of 40 nm, 100 nm, and 120 nm, using the same etch depths of 40 %, 60 %, 80 %, and 100 %. Simulations were conducted to assess the coupling efficiency spectra, revealing a trade-off between bandwidth and coupling efficiency. As representatives, three fully-etched grating couplers optimized for target bandwidths of 40 nm, 100 nm, and 120 nm were fabricated and tested. Compared to the simulation results, the measured coupling efficiencies showed a discrepancy of less than 0.5 dB, and the 3 dB bandwidths were also highly consistent, with a variance of only 7–19 nm.

Yoon et al. utilized a PSO-based inverse design approach to design a vertical emitting GC [99]. Unlike previous studies [98], [101], which focused on optimizing the grating period to achieve apodization, this study concentrated on optimizing the depth of each grating. The optimized GC was fabricated using a triple-etched meta-grating structure with three distinct height levels: 0 nm, 150 nm, and 220 nm. Simulation results indicated a coupling efficiency of −2.2 dB with a 3-dB bandwidth of 88 nm, however, experimental results revealed a performance degradation with a coupling efficiency of −4.2 dB with a 3-dB bandwidth of 48 nm. This performance degradation was attributed to fabrication-related issues, including undesired etching, over-etching due to mask misalignment, and variations in the sidewall angles. Hammond et al. applied a density-based TO method to design both single- and dual-polarization vertical emitting GCs on the double-layer platform [100]. This method optimized the full 3D structure across both SOI and Poly-Si layers to maximize coupling efficiency. The single-polarization GC showed coupling efficiency of −3 dB and a 3-dB bandwidth of 73 nm in simulation, but experimental results showed −4.7 dB with a 75 nm bandwidth, likely due to fabrication challenges such as conformal layer misalignment. The dual-polarization GC similarly showed a slight coupling efficiency drop from −5.6 dB (simulation) to −7 dB (experiment) though it maintained high polarization extinction. Despite the fabrication-related variations, both GCs showed consistent performance with a variation of only 2.4 dB across multiple wafers.

Although the fabrication and experimental validation of an inverse-designed GC were not provided, there exists research leveraging inverse design to demonstrate GCs with record-breaking performance. Michaels et al. demonstrated, through simulation, a grating coupler achieving a remarkable chip-to-fiber coupling efficiency of 99.2 % at a wavelength of 1,550 nm, with a 1-dB bandwidth of 24 nm [102]. This study presents an idealized design, highlighting the potential of inverse-designed grating couplers while setting a benchmark for ultra-high-efficiency GCs. Also, some studies have explored deep learning-based inverse design methods to enhance the computational efficiency, particularly for GC designs that typically require a large simulation cost. Tu et al. proposed a DNN-based GC optimization method with a data-driven approach to predict GC performance and also conduct inverse design [103]. In this work, two deep learning models were designed: a forward design model and an inverse design model. The forward design approach predicts a GC’s coupling efficiency and center wavelength based on input parameters such as the grating’s etching depth, pitch, and duty cycle. Conversely, the inverse design approach predicts GC design parameters from given values of coupling efficiency and center wavelength. A total of 937 datasets were gathered and utilized for training. The forward design model achieved a prediction accuracy of 91.7 % with a low MSE loss of 0.0034, while the inverse design approach also successfully generated viable designs with a reasonable MSE loss of 0.0403, closely matching the target spectrum. Witt et al. also explored the design and optimization of GCs using the deep learning method [65]. The fab-in-the-loop reinforcement learning approach, which incorporates feedback from real-world fabrication, was utilized along with the Deep Deterministic Policy Gradient (DDPG) algorithm. This method involved running 10,000 episodes to optimize 12 adjustable parameters with sub-wavelength holes, resulting in an optimized GC with an insertion loss of 3.24 dB, a marked improvement over the 8.8 dB loss seen with conventional methods. A summary of the results of these inverse-designed GCs is provided in Table 3.

**Table 3:** Summary of inverse-designed grating couplers.

Ref	Design method	Structure	CE (%) (sim./exp.)	3-dB BW (nm)
[91]	Forward	Chirped	42/34	48
[92]	Forward	Dual-etched	45.3/27.6	68
[95]	Recursive	Apodized grating	61.4/–	–/–
[96]	GA	Apodized grating	61/–	35/– (1-dB)
[97]	GA	Apodized grating	64.7/–	33/– (1-dB)
[98]	Gradient-based	Partial-etched binary grating	50.1/25.7	29/37
[99]	PSO	Triple-etched meta-grating	60.2/38	88/74
[100]	DTO	Double-layer (single pol.)	50.1/33.9	73/75
[101]	Gradient-based	Fully-etched binary grating	38/34.6	40/33
[102]	Gradient-based	Double-layer fully etched grating	99.2/–	24/– (1-dB)
[103]	DNN	Partial-etched binary grating	34.6/–	107/–
[65]	DDPG	Ph.C-based binary grating	47.4/47.4	29/

### 3.4 Waveguide devices: bends, crossings, and cavities

**Figure 12:**
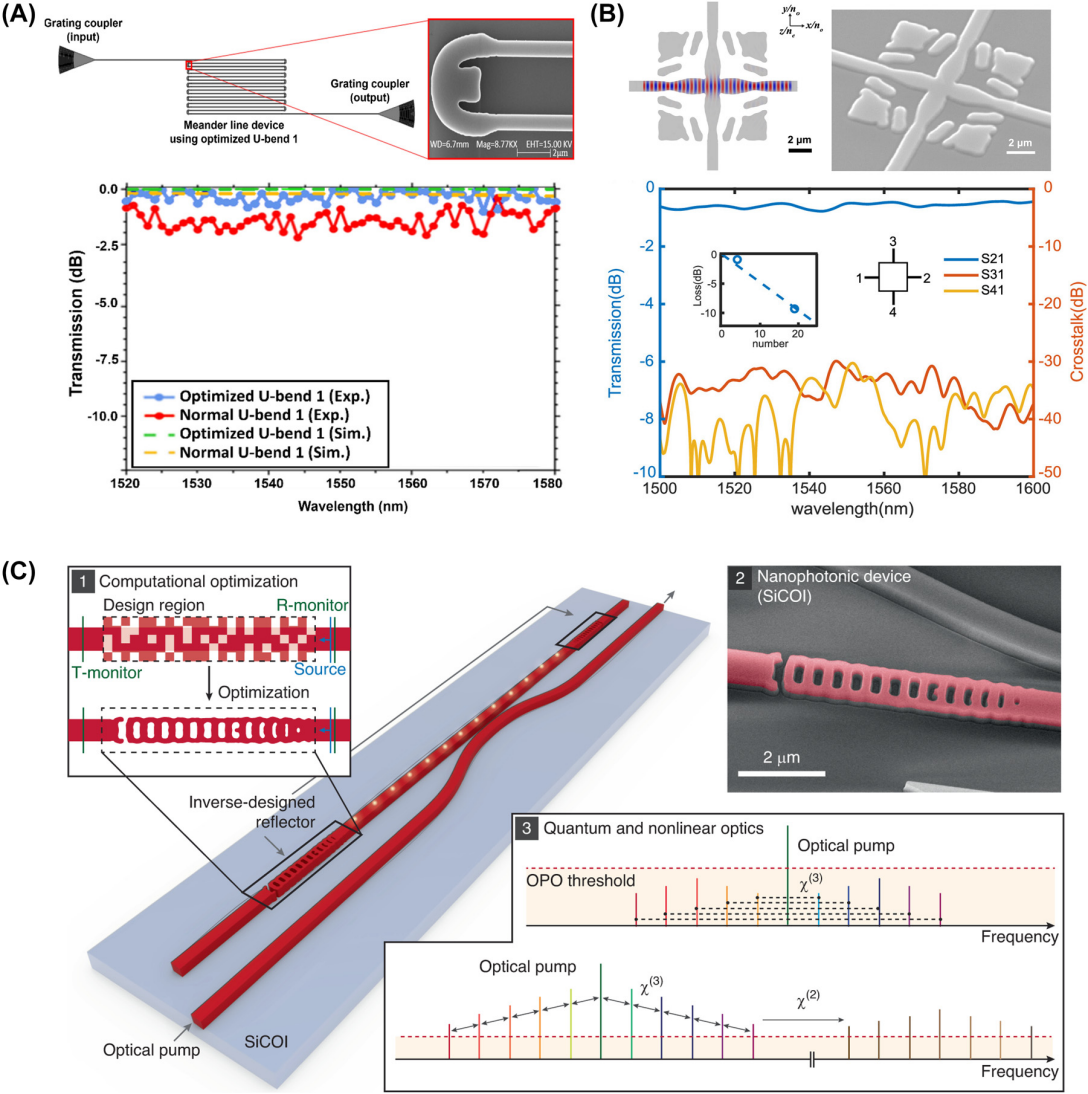
Representative examples of the waveguide devices. (A) Experimental setup and the SEM image of the fabricated U-bend device (up). Comparison graph between optimized and unoptimized U-bends in terms of experimental and simulation results (down). (B) SEM image of the TO-based waveguide crossing device on LNOI platform (up) and the obtained experimental results (down). (C) Optimization schematic of the reflector and the targeted comb response of the cavity (right). (A) Is reprinted from Ref. [104], with permission. Copyright 2024 American Chemical Society; (B) is reprinted from Ref. [105], with permission. Copyright 2024 Elsevier; (C) is reprinted from Ref. [106], with permission (CC BY 4.0).

Waveguide devices, fundamental components in nanophotonic circuits, are structures that confine and guide light along defined paths, enabling a wide range of optical functionalities on a compact scale. Inverse design techniques have revolutionized the development of these devices, allowing for the precise control of light propagation, coupling, and interaction with more compact and efficient devices, with representative examples shown in Figure 12.

Waveguide bends, crossings, and cavities are some of the simple yet crucial elements of photonic circuits. Conventional bends require adiabatic mode converters and a wide radius of curvature to evade bending loss and modal mismatch [107], [108]. To further reduce the bending curvature and avoid the implementation of mode converters, researchers have applied inverse design methods to design compact and efficient waveguide bends. Zhang et al. used PSO to design compact, and low-loss waveguide bends along with experimental verification [109]. Their design employed cubic spline interpolation optimized with PSO to achieve the most suitable bending shape that supports a targeted number of modes. The reported waveguide bend devices show insertion loss of 0.009 dB, 0.028 dB, and 0.048 dB for waveguides supporting one, two, and three modes respectively. In a recent study by Irfan et al., it was demonstrated that by using TO, it is possible to achieve compact and low-loss L-bend and U-bend structures for the SOI platform [104]. They reported three different bends for both shapes and with the help of TO, the insertion loss was reduced by nearly half. Waveguide bends, crossings and other types of devices have also been proposed on the lithium niobate-on-insulator (LNOI) platform, owing to the promising material characteristics of lithium niobate. In this regard, Shang et al. proposed inverse-designed mode multiplexers, waveguide crossings, and waveguide bend structures on the LNOI platform [105]. Their experimental results demonstrate that the bent waveguide reduces the insertion loss by up to 8 times compared to a waveguide bend with the same radius of curvature. All three devices operate between 1,500 nm and 1,600 nm with improved performance.

Beyond these fundamental elements of photonic circuits, advanced waveguide devices such as resonators, sensors, and gratings offer enhanced functionality and precision in light manipulation, paving the way for innovative applications in sensing, filtering, and signal processing. In this regard, Yang et al. proposed a silicon carbide (SiC)-based optical Fabry–Perot cavity design with the help of inverse-designed reflectors [106]. The proposed reflector had dimensions of 6.75 μm by 1 μm and represents one of the first demonstrations of inverse design applied to quantum and nonlinear light generation. Chung et al. proposed an inverse-designed waveguide biosensor operating at 1,550 nm on the SOI platform [110]. They implemented the high-contrast probe cleavage detection (HCCD) method to design and optimize the device, demonstrating a biosensor suitable for rapid sensing with a high transmission rate for the target molecule, thus enhancing sensing effectiveness and precision. The results of these inverse-designed waveguide devices are summarized in Table 4. 

**Table 4:** Summary of inverse-designed waveguide devices.

Ref	Design method	Waveguide device	Material platform
[107]	Forward	90° bend	SOI
[108]	Forward	90° bend	SOI
[109]	PSO	90° bend	SOI
[104]	TO	U-bend	SOI
[105]	TO	Waveguide crossing	LNOI
[106]	TO	Reflector	SiC

### 3.5 Metasurfaces

Metasurfaces, owing to their capability of manipulating wavefronts with sub-micron-thick optical elements, have attracted significant attention over the past decade. However, the extremely large degrees of freedom in their designs also present challenges in finding the optimized structure for a particular application. To address this problem, various inverse design methods, such as evolutionary algorithms, gradient-based methods, and deep learning methods have been utilized for the rapid design of high-performance metasurfaces. These inverse design methods can be categorized according to their main objective: reducing optimization iterations, accelerating forward calculations, and directly deriving structure from target properties without iteration. In this subsection, we introduce selected examples of inversely designed metasurfaces classified by their design methodology.

**Figure 13:**
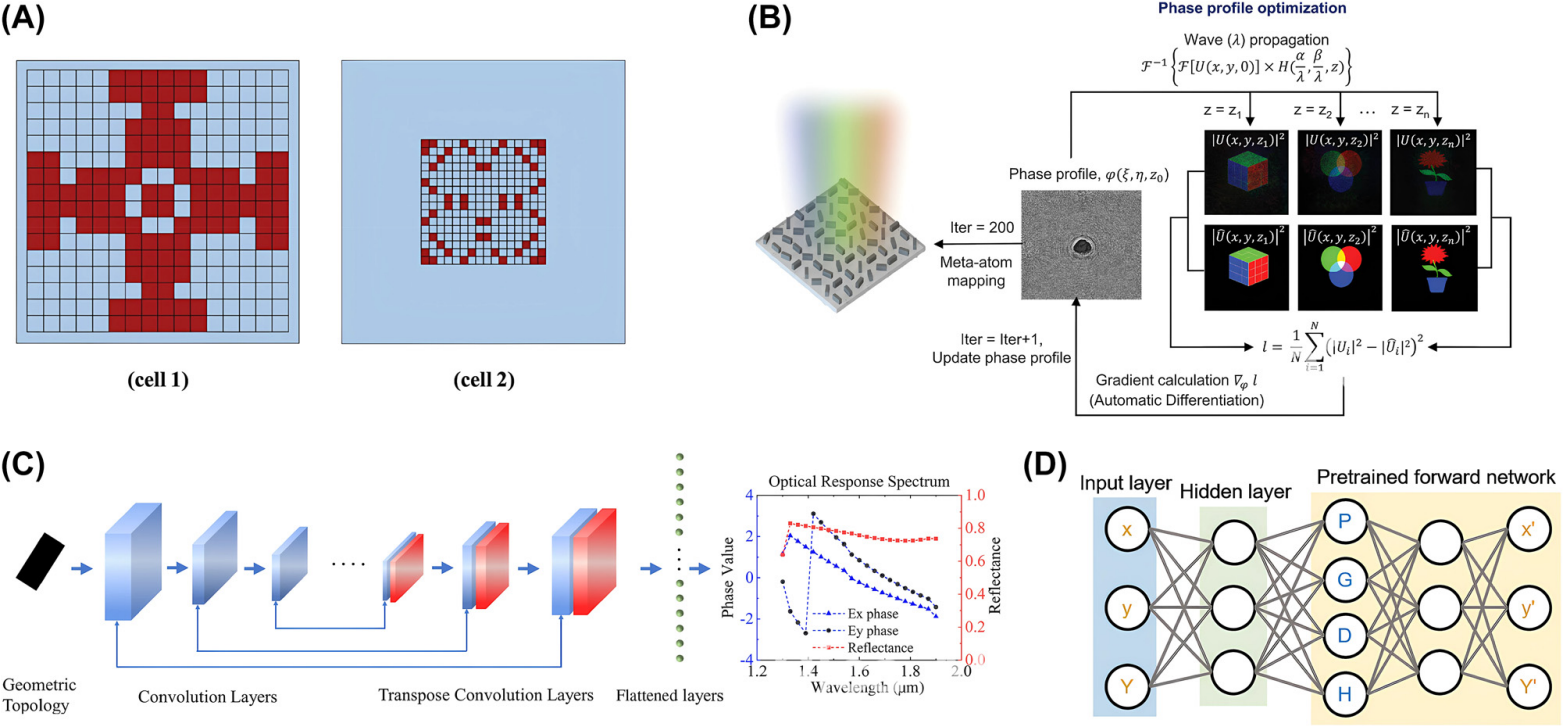
Representative examples of the inverse-designed metasurfaces. (A) Unit cell layouts optimized by binary PSO. (B) Flowchart of the inverse design method of multicolor hologram metasurfaces. (C) Network architecture of the physics-based neural network surrogate model. (D) Tandem DNN architecture used for silicon color prediction. (A) Is reprinted from Ref. [113], with permission (CC BY 4.0); (B) is reprinted from Ref. [114], with permission. Copyright 2024 John Wiley and Sons; (C) is reprinted from Ref. [115], with permission from Photonics Research; (D) is reprinted from Ref. [116], with permission. Copyright 2024 John Wiley and Sons.

Population-based heuristics and gradient-based methods have been utilized to minimize the design iterations needed to find the optimal structure. Sun et al. used a genetic algorithm to determine the optimal arrangement of “0” and “1” unit cells on the metasurface, achieving uniform backscattering throughout a broad frequency range with a reduced radar cross-section [111]. Work by Fan et al. utilized a genetic algorithm for the optimization of a 1D Pancharatnam–Berry phase-controlled metasurface [112]. The authors implemented a light-sheet mode by selecting appropriate phase profiles for unit cells. Haji-Ahmadi et al. designed a pixelated checkerboard metasurface for broadband radar cross-section reduction using a binary PSO algorithm [113]. A sigmoid limiting transformation was applied to the particle for binarization. The optimized unit cells are shown in Figure 13(A). The out-of-phase reflection from these unit cells enables broadband radar cross-section reduction.

Gradient-based optimization methods, including the adjoint method, have been widely implemented for the inverse design of metasurfaces [114], [117], [118], [119], [120]. In the work of Chung et al., a metalens with extremely high NA was theoretically demonstrated using an adjoint method and minimax optimization [118]. The designed metalens has a 2D freeform topology consisting of binary pixels. In another work by Chung et al. [119], adjoint-based local optimization was merged within a global optimization process for the design of a tunable beam-deflecting metasurface. The optimized triple grating structure achieved 80 % switching efficiency and a deflection angle of up to 144°. Mansouree et al. demonstrated a multifunctional 2.5D metasurface that focuses 2 different wavelengths at different focal points using adjoint optimization [120]. The authors illustrated that the inverse-designed structure outperforms the conventional unit-cell-based metasurfaces. Moreover, they showed that increasing the degree of freedom can further enhance the performance of multifunctional metasurfaces. So et al. designed multicolor and multiplane holograms enabled by single-cell metasurfaces [114]. A gradient descent optimization was implemented for the optimization of the phase profile for multiple wavelengths as shown in Figure 13(B). Automatic diffraction was used for the efficient calculation of gradients.

On the other hand, attempts have been made to accelerate forward calculations by replacing the conventional electromagnetic simulations with faster alternatives. Machine learning-based surrogate models and coupled mode theory (CMT) are examples. Jing et al. designed an orbital angular momentum multiplexing metasurface using an iterative hybrid optimization algorithm, which includes a neural-net surrogate model and genetic algorithm [115]. In the optimization process, the fitness of the structure was predicted using a physics-based neural net surrogate model. At the end of each iteration, genetic operators were applied for the reproduction of the next population. Wiecha et al. trained a CNN capable of predicting the near fields and far fields of a nanostructure [121]. In the work of Tanriover et al., a forward-predicting network consisting of an autoencoder and a fully connected network was used for the prediction of the optical response of metasurface unit cells [122]. Zhou et al. used coupled mode theory and adjoint optimization for the inverse design of large-area high NA metalenses [123]. The far-fields calculated by CMT were optimized with the adjoint method and then converted to precalculated geometric parameters. Wu et al. employed spatial coupled-mode theory for the inverse design of a large NA metalens [124]. By modelling the meta-atoms as truncated waveguides, they proposed an inverse design framework that is faster than conventional full-wave simulation.

Potentially, the fastest inverse design methods are those that can generate optimal structures directly from target properties. Liu et al. trained a generative adversarial network that generates the unit cell structure for a given transmittance spectrum [125]. Gao et al. addressed the non-uniqueness problem in inverse design by employing a bidirectional DNN with a tandem network architecture for the structural color design of silicon [116]. An inverse network was connected to a pre-trained forward network trained to predict the color of a given geometry. The tandem network was then trained to minimize the error between the input and output color. Lin et al. designed a nanodisc structure-based plasmonic metasurface using a CNN [126]. The CNN takes the absorption spectra as inputs and outputs the corresponding geometric parameters. The geometrical parameters were restricted to avoid non-uniqueness problems from symmetry. Jiang et al. presented a conditional generative neural network capable of global optimization for the inverse design of metagratings [127]. Forward and adjoint simulations were performed, providing physics-based gradients to the network. Chang et al. reported an inverse design method using factorization of Jones matrices [128]. The authors proved that an arbitrary Jones matrix could be implemented with a bilayer elliptical silicon nanopost array. Inverse-designed metasurfaces with 2 independent vectorial holographic images and an optical CNOT gate were demonstrated. The results of the inverse-designed metasurfaces are summarized in Table 5.

**Table 5:** Summary of inverse-designed metasurfaces.

Ref	Design method	Functionality	Note
[129]	Forward	Holograms	
[130]	Forward	Eyepiece for augmented reality	
[111]	GA	RCS reduction	Broadband, broad angle
[112]	GA	Light sheet mode	
[113]	BPSO	RCS reduction	Broadband
[118]	Adjoint	High-NA metalens (0.99)	Freeform
[119]	Adjoint + PSO	Tunable beam deflector (144°)	
[120]	Adjoint	Metalens	Multifunctional
[114]	Gradient descent	Multicolor/multiplane hologram	
[115]	NNSM + GA	OAM (de)multiplexer	
[122]	Autoencoder + FCN	Metalens	Fabrication feasible
[123]	CMT + Adjoint	Metalens, hologram	Large area
[124]	SCMT	Metalens, aberration-reduced lens	
[125]	GAN	Spectrum prediction	
[116]	Tandem network	Si structural color	
[126]	CNN	Plasmonic metasurface	
[127]	GLOnet	Metagrating	Global optimization
[128]	Matrix factorization	Vectorial hologram, CNOT gate	

## 4 Commercial and open-source inverse design tools

The optimization and inverse design techniques discussed so far rely on full-wave simulations, such as finite-difference time-domain (FDTD) and finite element method (FEM). FDTD solves the time-domain Maxwell equations on a structured Yee grid with a finite time step, while FEM does the time-harmonic Maxwell equations on a complex mesh using a weak-form discretization of linear equations. Many electromagnetic solvers now offer optimization plug-ins, allowing seamless integration of photonic simulations with post-simulation optimization workflows.

We note that, although there is no theoretical preference between these solvers once a certain level of accuracy is achieved, FDTD has recently been widely used for inverse design problems due to its ease of parallel computation and hardware acceleration. The Yee grid’s simple structure supports spatial parallelism across multiple processors, and the time-domain approach allows for broadband simulations in a single run, enabling frequency parallelism [131]. FDTD also benefits significantly from GPU acceleration, overcoming CPU memory bandwidth limitations [132].

In the following, we introduce several commercial and open-source tools commonly used in photonic inverse-design applications.

First, MEEP [131], [133] and Tidy3D [134] are open-source and commercial FDTD solvers, respectively, both available through Python interfaces. Users can define electromagnetic problems using Python scripts to set object geometries, material properties, wave sources, monitors, boundary conditions, and other parameters. Running these scripts generates EM field data and their derivatives such as the Poynting vector on the predefined monitors. Notably, MEEP, as a free and open-source tool, supports MPI-based parallel computing, while Tidy3D, built for GPU acceleration, is particularly efficient for large-scale problems with more than one billion grid points.

These tools support built-in adjoint-based optimization, which are integrated with Autograd [135] and JAX [136]. In this approach, a single optimization step involves running two electromagnetic simulations (forward and adjoint) to compute the gradient of the objective function with respect to optical parameters (permittivity). These automatic differentiation tools streamline the optimization process, from pre-simulation parameter adjustments that enforce fabrication constraints to post-simulation customization of the objective function using basic field elements. In addition, heuristic optimization techniques such as genetic algorithms and particle swarm optimization can be implemented with open-source libraries, such as pyGAD [137] and PySwarms [138].

In addition to the tools mentioned, Lumerical [139], a widely used photonics simulation software suite developed by Ansys, provides robust support for optimization workflows, particularly within its Lumerical FDTD Solutions. It includes built-in heuristic methods like PSO and supports adjoint method-based TO through Lumopt [140], a specialized Python API. Moreover, Lumerical is also compatible with external Python libraries, such as Splayout [141], allowing users to implement various heuristic algorithms like GA, PSO, DBS, as well as adjoint-based TO.

On the other hand, *COMSOL Multiphysics* [142], a commercial FEM solver, is also used for photonic inverse design. Unlike FDTD, FEM can address explicit material boundaries, which is particularly advantageous in cases involving sharp metallic object corners where electromagnetic fields are highly enhanced. Also, FEM can be computationally efficient for strongly resonant structures, which might take a long simulation time with FDTD. Consequently, FEM solvers, including COMSOL, are often a more suitable choice for inverse design problems, particularly those involving plasmonic objects [143]. Like FDTD solvers, COMSOL supports the MATLAB interface, allowing for optimizations such as particle swarm optimization [144] and adjoint sensitivity [44]. Moreover, TO codes in MATLAB, such as the one introduced by Ref. [145], can serve as a useful platform for implementing customized photonic design workflows within COMSOL.

Notably, simpler structures with specific symmetry may not require full-wave simulations. For example, RCWA [146], [147] is a semi-analytical method that efficiently solves Maxwell’s equations in Fourier space, especially for periodic structures. Similarly, infinite multilayer structures can be analyzed using the transfer matrix method (TMM) [148], [149], which relates the electromagnetic fields across the boundaries of different layers. Mie theory [150], [151] can solve problems involving cylindrical structures with radial symmetry. These methods are implemented in commercial software (e.g. Lumerical) and open-source platforms (e.g. RETICOLO), or can be developed in-house for heuristic or adjoint optimization, as well as for generating ground-truth data for the training of deep neural networks.

## 5 Commercial foundry implementations for silicon photonics and other material platforms

Most inverse designs have been fabricated primarily using electron-beam (e-beam) lithography, which enables reduced pixel sizes for optimal designs with an enhanced degree of freedom. While e-beam lithography is convenient for readily verifying the design feasibility through in-house fabrication, it remains inherently impractical for mass production. Therefore, the adoption of commercial foundry production utilizing deep ultraviolet photolithography (DUVL) is essential for the widespread dissemination of the inverse design in legacy PIC technologies. This section presents a range of examples where inverse-designed photonic devices have been successfully produced by DUVL (see Table 6), demonstrating how it bridges the gap between innovative designs and scalable manufacturing.

**Table 6:** Summary of silicon photonic devices fabricated using DUVL.

Ref.	Exposure wavelength	Method	Device	Minimum feature size	Foundry
[70]	248 nm	PSO	MMI	100 nm	IME
[152]	248 nm	ADJ	Crossing	500 nm	Sandia
[99]	248 nm	PSO	GC	200 nm	–
[153]	193 nm	PSO-ADJ	MC	200 nm	–
[154]	193 nm	ADJ	GC	65 nm	CEA-Leti
[155]	193 nm	ADJ	GC	60 nm	CEA-Leti
[156]	193 nm	ADJ-LST	GC-WDM	160 nm	AMF
[157]	193 nm	DTO	MDM, WDM, DC, BS	40 nm	AIM photonics
[100]	193 nm	DTO	GC	100 nm	Global foundries
[44]	–	DTO	Vector matrix product	–	AMF

Several research efforts in inverse design have leveraged DUVL with KrF [70], [99], [152] and ArF [100], [153]–[157] laser exposure (as summarized in Table 1). Various devices including an MMI power splitter (PS), vertical grating coupler (GC) [99], and mode converter (MC) [153] have been inversely designed with the aid of PSO and fabricated by DUVL. The adjoint method and adjoint-inspired designs have demonstrated vertical GC with an SOI single layer [154] and SOI/SiN dual layer [155], fabricated by DUVL at CEA-Leti. More recently, DUVL on AMF has fabricated a GC demultiplexer designed by the adjoint method with a fast integral technique [156].

The device geometry can be optimized to be more fabricable by enforcing the level-set fabrication constraint, where a penalty term can be incorporated to facilitate the concurrent optimization of performance and fabricability [158]. Inverse-designed devices including MDM, WDM, DC, and PS have been demonstrated under the constraints of a minimum gap and minimum radius curvature by DUVL at AIM Photonics [157].

The density-based topology optimization (DTO) method has been successfully implemented for DUVL-based fabrication of inversely designed devices. Fabrication constraints concerning minimum area and minimum enclosed area can be implemented in conjunction with previously established constraints of minimum linewidth, line spacing, and curvature [159]. Based on this method, it has been experimentally shown that both single and dual polarization vertical GCs can be fabricated through DUVL at AMF [100]. More recently, the two-dimensional effective index approximation has been applied to fabricate vector-matrix products for *N* by *N* matrix, inverse-designed by DTO with a large computational domain, via DUVL on AMF. The DTO technique has been theoretically investigated to ensure DRC compliance using a conditional generator for feasible design and straight-through estimator [160].

While the Si-based photonic platforms, leveraging mature CMOS fabrication technologies, remain dominant in inverse design methodologies, they inherently possess limitations such as weak nonlinear optical properties and a narrow transparency window. These drawbacks have driven interest in alternative materials – such as III–V compounds [161], silicon carbide (SiC) [106], diamond [162], and lithium niobate (LN) [105], [163] – which offer unique advantages that can be further enhanced through advanced inverse design techniques. Notably, commercial fabrication processes for these materials are less available, prompting research teams to rely on in-house e-beam lithography for fabrication.

Nevertheless, significant efforts have been devoted to inverse-designed photonic devices on these alternative material platforms to overcome their inherent fabrication constraints that are more challenging than those of Si-based counterparts, especially for compact devices, while simultaneously achieving decent device performances (see Table 7). A gallium arsenide (GaAs)-based inverse-designed coupler [161] has been demonstrated with a novel sleeve and bulk fabrication methodology to overcome feature-size-dependent etch rates in the reactive-ion-etching (RIE) process. A fabrication-tolerant inverse-designed Fabry–Pérot (FP) cavity structure based on SiC [106] has been explored to realize second and third-order nonlinear light generation, optimized for low scattering loss and enhanced robustness against fabrication errors. Implementing inverse-designed components on diamond platforms presents significant challenges due to critical constraints in diamond nanofabrication technologies. An advanced optimization-based inverse design technique was proposed to provide the full parameter space of fabricable devices, as demonstrated with a vertical coupler [162]. LN-based devices face difficulties not only in achieving minimum feature sizes but also in substantial sidewall angles due to the physical etching process, which should be accounted for during inverse design optimization processes. A variety of inverse-designed LN devices [105], [163] have been demonstrated to address these practical fabrication constraints.

**Table 7:** Summary of fabricated inverse-designed devices in other material platforms.

Ref.	Material	Method	Device	Lithography	Minimum feature size
[161]	GaAs	Gradient-based	GC	e-beam	150 nm
[106]	SiC	Gradient-based	FP cavity	e-beam	120 nm
[162]	Diamond	–	Vertical coupler	e-beam	100 nm
[105]	LN (z-cut)	Gradient TO	Mode multiplexer, crossing, bend	e-beam	200 nm
[163]	LN (x-cut)	Adjoint-based gradient	Mode converter	e-beam	424 nm

## 6 Discussions

In this review, the vast potential of inverse design methods is highlighted through representative examples. Although these methods have already achieved remarkable results, there is still room for further improvement. Currently, inverse design in nanophotonics is restricted by several challenges. First, the resolution of the fabrication process restricts the degree of freedom in the inverse design process. Commercial foundries including DUV and e-beam lithography, have a minimum feature size of 40 nm. Recently, novel lithography techniques such as extreme ultraviolet (EUV) lithography have been developed and hold promising potential for improving inverse design capabilities. Other challenges involved the inverse design techniques themselves. Inverse design methods are sometimes ineffective and are not the best option for every design task. In the following section, we discuss the challenges of inverse design methods for two categories: optimization methods-based inverse design and deep learning-based inverse design. By highlighting these challenges, we can focus on design tasks where inverse design methods are most effective. Furthermore, applications of the inverse design have extended its effectiveness in other fields beyond nanophotonics and optics. Insights gained from these diverse applications can inspire further advances in these methodologies.

### 6.1 Challenges in optimization methods

In this section, several challenges in optimization methods enabled by meta-heuristic algorithms are discussed. Primarily, meta-heuristic algorithms suffer from being sensitive to initial conditions and optimization parameters, as well as from being time-consuming. We next examine how these limitations affect the optimization process regarding several aspects: multi-objective optimization and global optimization.

Multi-objective optimization problems deal with the optimization of multiple conflicting objectives simultaneously. Such problems can have various solutions and multi-objective algorithms are essential for such cases. As the objectives conflict with one another, focusing on one objective often leaves others unmet. Thus, multi-objective algorithms aim to identify a solution set that closely approximates these conflicting objectives so that all objectives are at least partially fulfilled. Gradient-based algorithms, genetic algorithms, and particle swarm optimization are among the most popular methods for multi-objective optimization. Even though these methods have resulted in compact and highly efficient devices, multi-objective optimization also has some limitations and difficulties. One of the main challenges of these algorithms is the need to set appropriate values for various parameters. These parameters significantly influence the performance, convergence, and quality of the solutions generated by the algorithm. Population size, crossover rate, mutation rate for genetic algorithms, swarm size, inertia weight, and other constants for PSO are some of the parameters that greatly affect the optimization performance. Another challenge is the computational cost. The optimization process in nanophotonics involves a vast parameter space, such as shape, size, and material properties. Evaluating the performance of potential solutions in this wide range is computationally intensive, especially when multiple objectives must be considered simultaneously. Scalability is another important issue in multi-objective optimization. As the number of decision variables increases, along with the number of objectives, evolutionary algorithms’ performance might decline. These issues have been addressed and researchers have tried to develop ideas and new algorithms to overcome such limitations [164], [165], [166].

Global optimization also remains a challenge. The optimization results of algorithms do not always lead to the global optimum and can easily become trapped in local optima. As discussed in previous sections, the performance of meta-heuristic algorithms is often sensitive to the parameters and initial conditions. The performance is strongly dependent on each selection and parameter. Consequently, designers often resort to iterative optimization processes, adjusting parameters in a trial-and-error manner to obtain near-optimal solutions. This repetitive adjustment can result in an inefficient and time-consuming design process, especially in the case of more complex, multidimensional problems. Furthermore, such sensitivity increases the likelihood of suboptimal solutions and can lead to higher computational costs. Thus, the need for robust strategies that enhance global search capabilities and reduce the reliance on parameter tuning remains critical to overcome these inefficiencies.

### 6.2 Challenges in deep learning methods

As widely covered in this review, the broadened interest in artificial intelligence has accelerated research in nanophotonics due to its unique characteristics. Once trained, AI can generate optimized designs quickly, making them ideal for real-time applications. Moreover, it can be applied to a range of related design problems, offering flexibility that conventional optimization algorithms lack. But still, a one-time cost simulation is needed to train the AI. As structures become more complex, more data is required. Although increasing the quantity of labeled data leads to improved network performance, a major drawback of this design approach is its significant computational expense, especially when applied to advanced AI models that require enormous datasets. This challenge becomes even more pronounced in fields like nanophotonics, where training neural networks demands an extensive number of electromagnetic simulations. These simulations are computationally intensive and highly time-consuming, making it difficult to efficiently train models without access to substantial computing resources. Consequently, while more data holds the promise of better performance, practical limitations related to computational power and time requirements present significant barriers to scaling up this method in cutting-edge applications. Recent studies try to find a way to reduce this one-time cost by leveraging advanced techniques such as generative AI or transfer learning. For example, in Ref. [57], a semi-supervised learning technique with the generative model was applied to reduce the one-time cost simulations. In Ref. [167], transfer learning was implemented to improve the efficacy of deep neural networks for electromagnetic metamaterials. Because these researches are very new, there is a high potential to reduce the computational cost further with the development of AI algorithms.

Another concern is that the power of deep learning is limited in nanophotonics; while it can successfully inverse design devices at a one-time computational cost, the performance of these devices cannot exceed the capabilities within the training dataset and can only mimic their performance. To inverse design nanophotonic devices with even higher performance, an additional optimization process is required, which diminishes the advantages of deep learning methods.

In the future, deep learning methods can be developed to improve the inverse design of nanophotonic devices. First, deep learning methods can design nanophotonic devices at the speed of AI, by reducing the time cost of electromagnetic simulations by implementing models like physics-informed neural networks. Second, the development of AI algorithms such as transfer learning, continuous learning, etc., and the development of models such as vision transformers, etc. can accelerate the inverse design capabilities, paving the way for designing high-performance nanophotonic devices.

### 6.3 Inverse design in other research fields

While inverse design has been predominantly developed for applications in nanophotonics and optics, it has increasingly demonstrated its versatility and effectiveness across a range of scientific domains. Its scope has broadened to include areas such as advanced photonic system design [168], metastructure engineering within the microwave spectrum [169], sophisticated mechanical metamaterial design for tailored properties [170], and the search of materials with target functionalities [171]. These advancements in related research fields not only highlight the inverse design method’s exceptional adaptability, but also offer valuable insights and methodologies, inspiring innovative solutions and transformative breakthroughs in nanophotonic and optical design.

## 7 Conclusions

In this review, we summarized the latest advancements in inverse design techniques applied to nanophotonics, which merge nanotechnology and photonics. The first section covers the inverse design algorithms utilized in the design of nanophotonic devices, discussing these methods in detail along with their theoretical backgrounds and representative examples. The second section presents a range of inverse-designed nanophotonic devices, categorized by their functionalities, showcasing the versatility of inverse design in creating diverse and high-performance devices. We also highlight open-source deep learning platforms that facilitate the inverse design process, as well as commercial foundries capable of fabricating these advanced devices. Finally, we explore the challenges that remain in inverse design methods, identifying key areas for future innovation.

Among the achievements in nanophotonic devices through various inverse design methods, there is still significant potential for further advancements. Current limitations in computational resources, fabrication feasibility, and design flexibility continue to pose challenges, but they also provide fertile ground for future exploration. Enhancing the efficiency of optimization algorithms, improving the accuracy of machine learning models for design prediction, and advancing nanofabrication technologies will be key factors in pushing the boundaries of what can be achieved with inverse design in nanophotonics. Additionally, as nanophotonic devices are increasingly adopted in real-world applications, new requirements and constraints will emerge. For example, designing devices that are robust against fabrication tolerances, environmental variations, and large-scale integration will become increasingly critical. Moreover, expanding the scope of inverse design to account for multi-physics optimizations, such as thermal and mechanical considerations, will allow for the development of more versatile and reliable devices. Overall, while the field of inverse-designed nanophotonics has already demonstrated remarkable potential, the road ahead is filled with opportunities for innovation. The future of nanophotonics, driven by intelligent design integrating inverse design and deep learning methods, holds the promise of transforming the way we manipulate and utilize light at the nanoscale.
